# D25V apolipoprotein C-III variant causes dominant hereditary systemic amyloidosis and confers cardiovascular protective lipoprotein profile

**DOI:** 10.1038/ncomms10353

**Published:** 2016-01-21

**Authors:** Sophie Valleix, Guglielmo Verona, Noémie Jourde-Chiche, Brigitte Nédelec, P. Patrizia Mangione, Frank Bridoux, Alain Mangé, Ahmet Dogan, Jean-Michel Goujon, Marie Lhomme, Carolane Dauteuille, Michèle Chabert, Riccardo Porcari, Christopher A. Waudby, Annalisa Relini, Philippa J. Talmud, Oleg Kovrov, Gunilla Olivecrona, Monica Stoppini, John Christodoulou, Philip N. Hawkins, Gilles Grateau, Marc Delpech, Anatol Kontush, Julian D. Gillmore, Athina D. Kalopissis, Vittorio Bellotti

**Affiliations:** 1 Université Paris-Descartes, Sorbonne Paris Cité, Assistance Publique-Hôpitaux de Paris, Laboratoire de Biologie et Génétique Moléculaire, Hôpital Cochin, Paris 75014, France; 2 INSERM, UMR_1163, Institut Imagine, Laboratoire de Génétique Ophtalmologique (LGO), Université Paris Descartes, Sorbonne Paris Cité, Paris 75015, France; 3 INSERM, U1016, Institut Cochin, Université Paris Descartes, Sorbonne Paris Cité, Paris 75014, France; 4 Sorbonne Universités, UPMC Univ Paris 06, INSERM, Université Paris-Descartes, Sorbonne Paris Cité, UMR_S 1138, Centre de Recherche des Cordeliers, Paris 75006, France; 5 Centre for Amyloidosis and Acute Phase Proteins, National Amyloidosis Centre, University College London, London NW3 2PF, UK; 6 Department of Molecular Medicine, Institute of Biochemistry, University of Pavia, Via Taramelli 3b, Pavia 27100, Italy; 7 Université de Marseille, AP-HM, Hôpital de la Conception, Marseille 13005, France; 8 Université de Poitiers, CHU Poitiers, Department of Nephrology and Kidney Transplantation, Centre National de Référence Amylose AL et autres maladies par dépôts d'immunoglobulines monoclonales, Poitiers 86021, France; 9 Institut de Recherche en Cancérologie de Montpellier (IRCM), Montpellier 34298, France; 10 INSERM, U1194, Montpellier 34298, France; 11 Université de Montpellier, Montpellier 34090, France; 12 Institut régional du Cancer de Montpellier, Montpellier 34298, France; 13 Department of Laboratory Medicine and Pathology, Mayo Clinic, Rochester, MN 55901, USA; 14 Departments of Laboratory Medicine and Pathology, Memorial Sloan-Kettering Cancer Centre, New York, NY 10065, USA; 15 Université de Poitiers, CHU Poitiers, Service d'Anatomie et Cytologie Pathologiques, Centre National de Référence Amylose AL et autres maladies par dépôts d'immunoglobulines monoclonales, Poitiers 86021, France; 16 Lipidomic core, ICANalytics, Institute of Cardiometabolism and Nutrition, ICAN, Pitié-Salpôtrière Hospital, F-75013 Paris, France; 17 Sorbonne Universités, UPMC Univ Paris 06, Institute of Cardiometabolism and Nutrition (ICAN), UMR_S 1166, Hôpital de la Pitié, Paris 75013, France; 18 Ecole Pratique des Hautes Etudes, PSL Research University, Laboratoire de Pharmacologie cellulaire et Moléculaire, Paris 75006, France; 19 Institute of Structural and Molecular Biology, University College London and Birkbeck College, University of London, London WC1E 6BT, UK; 20 Department of Physics, University of Genoa, Via Dodecaneso 33, Genoa 16146, Italy; 21 Centre for Cardiovascular Genetics, Institute of Cardiovascular Science, University College London, London WC1E 6JF, UK; 22 Department of Medical Biosciences, Umeå University, Umeå SE-901 87, Sweden; 23 Hôpital Tenon, AP-HP, Service de Médecine Interne, Centre de référence des amyloses d'origine inflammatoire et de la fièvre méditerranéenne familiale, Paris 75020, France

## Abstract

Apolipoprotein C-III deficiency provides cardiovascular protection, but apolipoprotein C-III is not known to be associated with human amyloidosis. Here we report a form of amyloidosis characterized by renal insufficiency caused by a new apolipoprotein C-III variant, D25V. Despite their uremic state, the D25V-carriers exhibit low triglyceride (TG) and apolipoprotein C-III levels, and low very-low-density lipoprotein (VLDL)/high high-density lipoprotein (HDL) profile. Amyloid fibrils comprise the D25V-variant only, showing that wild-type apolipoprotein C-III does not contribute to amyloid deposition *in vivo*. The mutation profoundly impacts helical structure stability of D25V-variant, which is remarkably fibrillogenic under physiological conditions *in vitro* producing typical amyloid fibrils in its lipid-free form. D25V apolipoprotein C-III is a new human amyloidogenic protein and the first conferring cardioprotection even in the unfavourable context of renal failure, extending the evidence for an important cardiovascular protective role of apolipoprotein C-III deficiency. Thus, fibrate therapy, which reduces hepatic *APOC3* transcription, may delay amyloid deposition in affected patients.

## Abstract (web summary)

Decrease in Apolipoprotein C-III (ApoC-III) yields a cardioprotective lipoprotein profile. Here, Valleix *et al.* reveal a novel ApoC-III variant conferring low plasma ApoC-III concentration and cardioprotection despite renal insufficiency, and, unexpectedly, causing dominant hereditary systemic amyloidosis due to its fibrillogenic nature.

## Graphical abstract

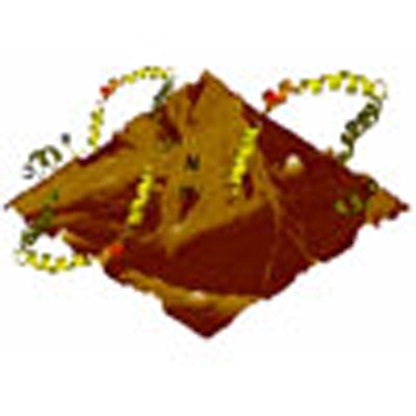

There is growing evidence that apolipoprotein C-III (apoC-III), a small exchangeable apolipoprotein carried in the circulation by VLDL and HDL^1^, is a master regulator of plasma TG homeostasis and downregulation of its expression has a cardiovasculo-protective effect^2^. ApoC-III inhibits the activity of lipoprotein lipase (LPL), the enzyme catalysing the first and rate-limiting step of TG-rich lipoprotein catabolism^3^, and also impairs uptake of TG-rich lipoprotein-remnants by the low-density lipoprotein (LDL)-receptor (LDL-R), increasing remnant residence time in the circulation^4^. ApoC-III is a glycosylated apolipoprotein composed of 79 amino acids, synthesized mainly in the liver, and existing in three isoforms, apoC-III_0_, apoC-III_1_ and apoC-III_2_ with, respectively, 0, 1 and 2 residues of sialic acid per molecule, but little is known about sialylation of apoC-III in disease states. Heterozygosity for the rare null allele of *APOC3,* R19X, has been associated with high levels of plasma HDL cholesterol (HDL-C), low levels of plasma TG and reduced incidence of ischaemic cardiovascular disease (CVD) in the Amish population^5^. Subsequently, genome-wide association studies also identified the Arg19Stop variant in other population isolates with a strong atheroprotective phenotype^6^. More importantly, a recent general population-based study confirmed the association of *APOC3* deficiency with an atheroprotective lipid profile and clinical protection against ischaemic CVD^7^,^8^.

In its lipid-bound state, apoC-III is composed of six amphipathic α-helices, the structural motif shared by all exchangeable apolipoproteins that confers their structural stability^9^. Conversely, apolipoproteins in lipid-free form have lower conformational stability and some, such as native and/or variants of apoA-I (ref. 10), apoA-II (ref. 11) and apoA-IV (ref. 12), are prone to self-assembly *in vitro* and *in vivo* as amyloid fibrils. Recombinant wild-type apoC-III forms insoluble aggregates with cross-β structure *in vitro*, reminiscent but not typical of amyloid fibrils^13^. Amyloid fibril formation is associated with a wide variety of diseases, and the hereditary monogenic forms of human amyloidoses, although rare, provide unique insight into mechanisms of protein misfolding and fibrillogenesis as recently highlighted by the discovery of the amyloidogenic variant of β2-microglobulin^14^.

Here we report a French family with severe renal amyloidosis and hypotriglyceridemia, both caused by a novel D25V apoC-III variant. Remarkably, this variant induced a favourable cardiovascular lipoprotein profile, even in the context of chronic kidney disease (CKD). The D25V protein is highly fibrillogenic in its lipid-free state, and forms amyloid fibrils under physiological conditions *in vitro*. This study provides new insights into the genetics of apoC-III and into VLDL and HDL metabolism in health and disease, illustrating the increasing importance of apoC-III in diverse biological processes.

## Results

### Patients

**Figure 1:**
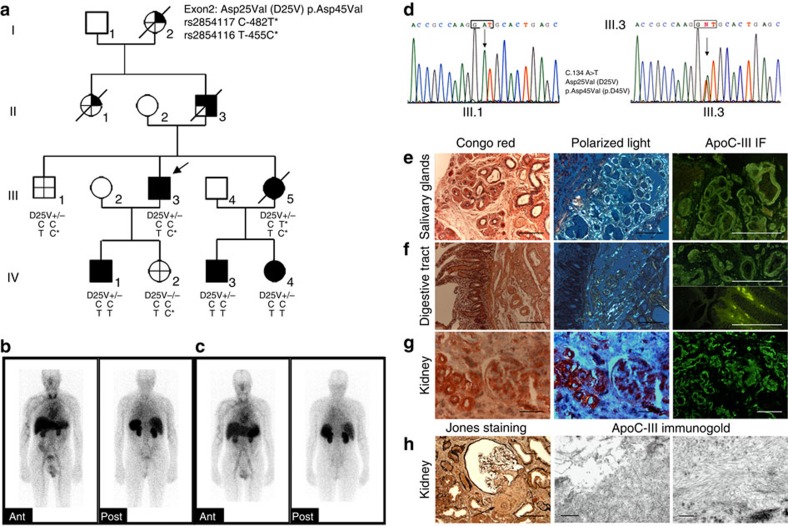
Apolipoprotein C-III amyloidosis in a French family. (**a**) Pedigree showing three generations of transmission of the *ApoC3* D25V variant with systemic amyloidosis and decreased plasma triglyceride levels: 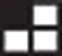
*APOC3* mutation present; 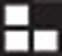 clinical syndrome; 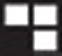 ApoC-III amyloid confirmed histologically; 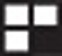 decreased plasma triglyceride levels; 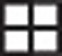 no clinical syndrome, no amyloid on histology, absence of *APOC3* mutation, no hypotriglyceridemia. *APOC3* haplotypes distribution of C-482T and T-455C polymorphisms is shown. D25V+/−, heterozygous for the apoC-III amyloidogenic variant; D25V−/−, absence of apoC-III mutation. (**b**,**c**) Serial anterior and posterior planar whole-body ^123^I- SAP scan. The scans from III.3 in **b** and IV.3 in **c** show a congested liver and amyloid in the spleen and kidneys. (**d**) Partial sequence chromatograms of *APOC3* exon 3. (**e**) Amyloid deposits in salivary glands from proband III.3. (**f**) Amyloid deposits in the duodenum from proband III.3 predominate in the submucosa around the vessels and around Brenner's glands. There are also amyloid deposits grouped in small clods within the intestinal villi. (**g**) Abundant vascular, moderate glomerular and interstitial amyloid deposits in kidney from proband III.3. For each tissue in **e**–**g**, left panels show the Congo red staining, scale bar, 100 μm; middle panels show the Congo red-stained section viewed under polarized light, scale bar, 100 μm; right panels show the immunofluorescence with anti-apoC-III antibody; scale bar, 100 μm. (**h**) Left panel: amyloid deposits in the kidney are observed in the mesangium and the glomerular arteriole (arrow) (Jones staining, scale bar, 100 μm); middle and right panels: electron micrograph of amyloid fibrils on immunogold staining with anti-apoC-III antibody, scale bar, 500 and 200 nm, respectively).

The proband (III.3 in Fig. 1a) presented at age 51 years with sicca syndrome and hypertension. Two years later, he was diagnosed with CKD; his serum creatinine level was 1.9 mg dl^−1^ with modest proteinuria (40 mg dl^−1^). Salivary gland and renal biopsies showed amyloid deposits. A search for an underlying monoclonal gammopathy was negative, and plasma concentrations of serum amyloid A protein (SAA) and C-reactive protein were in the normal range. The proband reached end-stage renal disease (ESRD) aged 56 years and received a cadaveric kidney transplant uneventfully. ^123^I-labelled serum amyloid P component (SAP) scintigraphy, undertaken just before renal transplantation in the proband III.3 (Fig. 1b) and in affected member IV.3 (Fig. 1c), showed a small total body amyloid load in the spleen and kidneys, at which point a fat aspirate demonstrated extensive amyloid. Four years after renal transplantation, graft function was normal (serum creatinine 0.88 mg dl^−1^), without detectable proteinuria. There was a strong family history of amyloidosis (Fig. 1a) with all affected members suffering first from sicca syndrome and later developing mildly proteinuric CKD. The proband's grandmother (patient I.2) and aunt (patient II.1) had suffered from sicca syndrome for many years before developing systemic amyloidosis and had died aged 74 and 80 years, respectively. The proband's father (patient II.3) had presented with sicca syndrome aged 63 years, Raynaud phenomenon, high blood pressure and diffuse pruritus. Salivary gland, rectal and bronchial biopsies had all shown amyloid. He reached ESRD aged 64 years. The proband's younger sister (patient III.5) had died aged 49 years of staphylococcal infection, shortly after kidney transplantation. She had presented with cardiac amyloidosis proven by biopsy 10 years earlier, associated with CKD and mild proteinuria. She had reached ESRD and started haemodialysis aged 40 years. She had also suffered from sicca syndrome, Raynaud phenomenon, oesophageal reflux with oesophagitis, diffuse pruritus and severe hypertension. Her two children (patients IV.3 and IV.4) presented with sicca syndrome at age 23 and 20 years, respectively, and both of them showed amyloid deposits on salivary gland biopsies (Supplementary Fig. 1). Both of them subsequently developed hypertension and stage II CKD. The proband's son (IV.1) suffered from sicca syndrome aged 30 years and showed amyloid deposition on salivary gland biopsy (Supplementary Fig. 1), but he had no hypertension and his renal function was normal with a serum creatinine level at 0.73 mg dl^−1^ and no proteinuria. The proband's daughter (IV.2) was clinically asymptomatic, and had no amyloid deposits on salivary gland biopsy.

### Amyloid and hypotriglyceridemia in D25V apoC-III carriers

An initial genetic screening for mutations in all genes recognized until now to be linked to hereditary amyloidosis was negative (see Methods for details). Based on the fact that affected patients with amyloidosis also displayed hypotriglyceridemia and increased plasma levels of HDL-C as compared with non-carriers in the family (Table 1), we next screened several candidate genes including the *APOC3*, *APOC1, APOC2, APOC4*, *APOA4*, *APOA5* and *APOE* genes. The unique variant identified was a single-base substitution (c.134A>T) in exon 3 of *APOC3*, encoding replacement of the negatively charged aspartate residue at position 25 of the mature apoC-III by a valine residue (D25V; Fig. 1d). All D25V-carriers displayed a 30–50% decrease in plasma apoC-III concentration (Table 1), and there was no correlation between *APOC3* promoter polymorphism genotypes and plasma TG levels among family members (Fig. 1a). Therefore, D25V apoC-III variant was uniquely found in family members affected with amyloidosis, hypotriglyceridemia and reduced apoC-III levels, and not in healthy normotriglycemic family members, demonstrating the co-inheritance of this variant with both the lipidemic and amyloidogenic phenotypic traits.

### Histology and LMD/MS-proteomic analysis of amyloid deposits

To establish that apoC-III D25V variant is the causative amyloidogenic protein, we analysed amyloid deposits by immunohistochemical staining, immunogold technology and proteomic methodologies. Amyloid deposits were discovered in a total of 17 diverse disease tissues from 6 different affected family members through three generations (kidney, salivary glands, heart, bowel, fat, and bronchia from III.3; kidney, salivary glands, skin, heart, liver from III.5; salivary glands, kidney, skin, kidney, bowel from II.3; salivary glands from IV.1, IV.3 and IV.4). All patients with sicca syndrome, the first clinical symptom, were carriers of D25V (II.3, III.3, III.5, IV.1, IV.3 and IV.4) and had amyloid deposits in salivary gland biopsies. Conversely, clinically non-affected individuals and non-carriers of D25V (III.1 and IV.2) demonstrated no amyloid deposits on salivary gland biospsies. In the proband III.3, all amyloid deposits observed in salivary gland, digestive tract and kidney were Congo red positive and immunoreactive with the anti-apoC-III antibody (Fig. 1e–g). Moreover, extensive amyloid deposits were found around salivary acini, in the interstitial matrix and in the walls of blood vessels, and were associated with areas of fibrosis and massive acinar atrophy (Fig. 1e). Similar pathological findings in salivary glands from younger affected family D25V-carriers (individuals IV.1, IV.3 and IV.4) were found and are shown in Supplementary Fig. 1. Therefore, this extensive amyloid accumulation associated with gland atrophy could explain the sicca symptoms of mouth reported by the patients. Renal histology from affected proband III.3 at an advanced stage of renal insufficiency showed diffuse amyloid deposition in the renal cortex that mostly affected the vascular compartment with abundant amyloid deposits in the walls of renal arterioles leading to lumen obliteration (Fig. 1g,h). Deposits were also observed in the glomeruli with prominent mesangial distribution, within the renal interstitium, and in the peritubular basement membranes with tubular atrophy. Amyloid P component co-localized with the above-mentioned deposits, and vascular, glomerular, tubular and interstitial amyloid deposits were positively and specifically immunoreactive with apoC-III antibody (Fig. 1g). Electron micrographs of the kidney specimen from III.3 showed numerous non-branched and randomly arranged deposits with a fibrillar appearance, measuring 7–10 nm in diameter, typical of amyloid (Fig. 1h). Immunoelectron microscopic analysis subsequently showed that these renal fibrillar aggregates were specifically labelled by gold-conjugated anti-apoC-III antibody (Fig. 1h). Therefore, our histologic analysis, in conjunction with ^123^I-SAP scintigraphy, clearly established the correlation between the clinical symptoms of D25V-carriers (sicca syndrome and renal failure) and the presence of amyloid in the respective tissues. In all 11 affected tissues from 6 D25V-carriers over three generations, the anti-apoC-III antibody consistently and specifically bound to all Congo red-staining amyloid areas, whereas there was no binding of antibodies against the other known amyloid fibril proteins. Moreover, the fact that anti-apoC-III antibody directly bound to the fibrillar aggregates confirms at the ultrastructural level the diagnosis of apoC-III amyloidosis.

**Figure 2:**
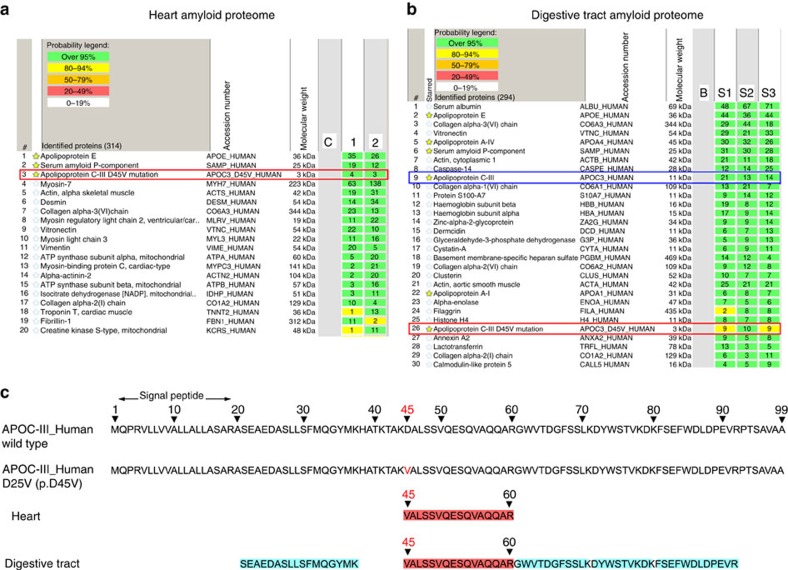
Proteomic analysis of amyloid plaques. Proteomic analyses of amyloid plaques from heart (**a**) and digestive tract (**b**) biopsy specimens of patient III.3 showing the most abundant proteins found. In both amyloid proteomes, the variant apoC-III protein was identified along with the apolipoprotein E and the serum amyloid P component. (**c**) ApoC-III tryptic peptide coverage: the two top lines represent the wild-type apoC-III sequence with the 20 amino-acid signal peptide and the mutant D25V (p. D45V) apoC-III sequence, respectively, and the two bottom lines represent the apoC-III peptides identified from the amyloid plaques from the heart and digestive tract, respectively. All amyloid plaques contained only the mutated apoC-III peptide with the valine at position 45 (corresponding to position 25 of the mature protein) and not the corresponding apoC-III wild-type peptide with the aspartate residue.

In addition to immunofluorescence and immunogold staining, amyloid subtyping was also confirmed by laser microdissection/mass spectrometry (LMD/MS)-based proteomics on three different tissues: digestive tract and heart from proband III.3, and salivary glands from individual IV.3, with several amyloid deposits microdissected per tissue (Fig. 2a,b and Supplementary Table 1). In all proteomes, only the variant apoC-III containing Valine 25 was invariably found in the diverse amyloid deposits along with SAP and apoE, with no trace of the corresponding wild-type apoC-III peptide (Fig. 2c). In the amyloid proteomes of the digestive tract and salivary glands, wild-type apoA-IV and apoA-I proteins were variably present, whereas both proteins were absent in heart proteome (Fig. 2a,b and Supplementary Table 1). These findings are in agreement with a number of studies showing the presence of apoA-I and A-IV in various amyloid deposits, irrespective of the nature of the amyloidogenic protein^15^. The concordance between the amyloid proteomes from different tissues of three D25V-carriers of this family, and the fact that no other amyloid-associated proteins were found in the heart except the mutated apoC-III clearly demonstrated that the mutated apoC-III was the amyloid fibril protein. Importantly, neither peptides representing the D25V or the wild-type apoC-III have been identified in the amyloid proteomes from over 5,000 patients with different types of systemic amyloidosis^15^, clearly indicating that apoC-III is not a protein commonly co-deposited in amyloid aggregates. Therefore, the D25V variant was specifically found in this kindred for the first time, and histologic and proteomic analyses were consistent with the genetic findings.

### MS analysis of apoC-III from D25V-HDLs

**Figure 3:**
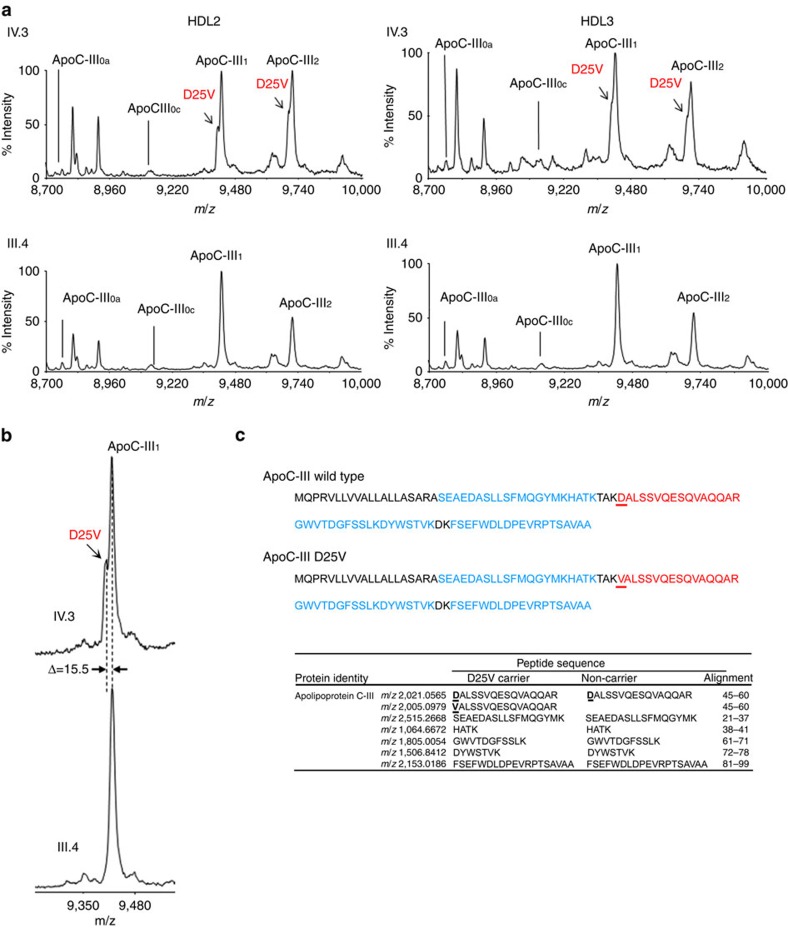
MS characterization of wild-type and D25V apoC-III isoforms in HDL2 and HDL3. (**a**) MS analysis of apoC-III isoforms from HDL2/HDL3. Representative graphs of apoC-III isoforms from D25V carrier IV.3 and non-carrier III.4 are shown. Three apoC-III isoforms are commonly referred to as apoC-III_0_, apoC-III_1_ and apoC-III_2_, where the index represents the number of sialic acid residues in each isoform. The subscript lower case letter indicates the absence of glycans (apoC-III_0a_) or the presence of a GalNAc-Gal disaccharide (apoC-III_0c_), respectively. The arrow points to the peak corresponding to the D25V variant of apoC-III isoforms with a reduced mass of 15,500 Da (apoC-III_1_: 9,406.11; apoC-III_2_: 9,708.90) in comparison to the wild-type apoC-III isoforms (apoC-III_1_: 9,421.61; apoC-III_2_: 9,714.40). The variant apoC-III_1_ and apoC-III_2_ isoforms are detected only in D25V-carriers. (**b**) Higher magnification of apoC-III_1_ from the D25V-carrier IV.3 and a non-carrier III.4. (**c**) Mass spectrometric identification of apoC-III in HDL2/3 using LC-MS/MS sequencing. The amino-acid sequences obtained using MS/MS are in bold. Specific wild-type or mutated peptides are in red. The observed *m/z* of peptides (including variable modifications) are listed in the table with their position in the apoC-III protein sequence.

To determine the relative amounts of wild-type and mutant apoC-III as well as the proportions of apoC-III isoforms in HDL2 and HDL3, MS was carried out in these lipoproteins from hypotriglyceridemic D25V carriers III.3, IV.3 and IV.4 and normotriglyceridemic non-D25V-carrier III.4. The resulting MS profiles showed that D25V-carriers exhibited a double peak for apoC-III_1_ and apoC-III_2_ isoforms (only the profile of individual IV.3 is presented), whereas the non-carrier of D25V had a normal profile (Fig. 3a). The measured mass difference of 15.5 Da between the two signals is in agreement with the difference between the wild-type aspartate residue and the mutated valine residue (15.9 Da). A higher magnification of MS profile is presented in Fig. 3b. To confirm that the additional peak observed in D25V-carriers corresponds to the apoC-III variant, LC/MS-MS sequencing was performed. As shown in Fig. 3c, both the tryptic peptides containing the mutant Val25 and the wild-type Asp25 of apoC-III isoforms were identified in heterozygous D25V-carriers, whereas individual III.4 exhibited the wild-type apoC-III tryptic peptide on HDL2/HDL3 subfractions.

Relative quantification of each apoC-III isoforms was performed in the three D25V-carriers (III.3, IV.3 and IV.4) and compared with the non-carrier individual III.4. Data, summarized in Table 2, show a twofold reduction of D25V-apoC-III compared with its wild-type counterpart in all three affected patients with amyloidosis. The levels of di-sialylated apoC-III were higher in D25Vcarriers compared with the non-D25V-carrier, with a corresponding decrease in mono- and non-sialylated apoC-III.

### Lipoprotein studies

**Figure 4:**
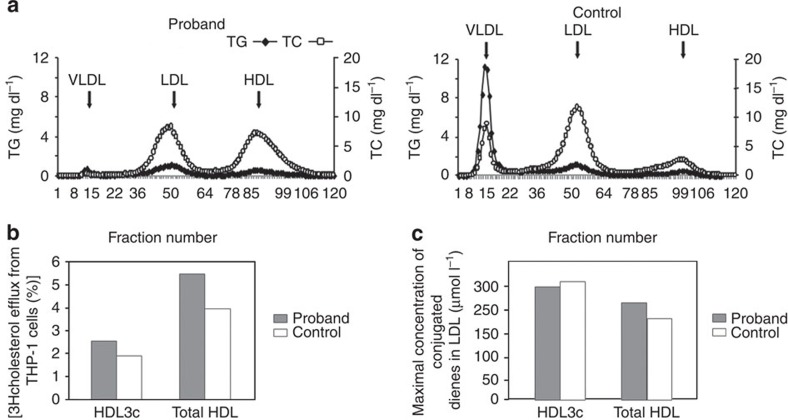
Lipoprotein studies. (**a**) Fast protein liquid chromatography (FPLC) size exclusion chromatogram of lipoproteins of III.3 in comparison to a normolipidemic control subject. In each case 200 μl of serum was analyzed using two GE Healthcare 10/300 GL Superose 6 columns coupled in series, equilibrated and eluted with a buffer containing 0.15 M NaCl, 3 mM EDTA, 0.04% sodium azide, pH 7.4 at 0.4 ml min^−1^. Triglyceride (TG) and total cholesterol (TC) were determined in each fraction. (**b**) Cholesterol efflux capacity of HDL. THP-1 macrophages were cholesterol-loaded by incubation with [3H]cholesterol-labelled acetylated LDL (1 μCi ml^−1^) for 24 h as detailed in the Methods. Cellular cholesterol efflux to HDL3c and total HDL from III.3 and one normolipidemic control subject (15 μg ml^−1^ HDL-PL per well) was measured in serum-free medium for a 4 h chase period. The percentage of cholesterol efflux was calculated as (medium cpm)/(medium cpm+cell cpm) × 100%. Specific cholesterol efflux was determined by subtracting nonspecific cholesterol efflux occurring in the absence of cholesterol acceptors (HDL). (**c**) Antioxidative activity of HDL. The antioxidative capacity of HDL3c and total HDL from III.3 and one normolipidemic control subject to inhibit LDL oxidation was assayed at a physiological HDL to LDL ratio. HDL particles were compared on the basis of their total mass. Lipoprotein oxidation was induced by the azo-initiator 2,2′-azo-bis-(2-amidinopropane) hydrochloride (final concentration 1 mmol l^−1^). Oxidation was expressed as the concentration of lipid hydroperoxides with conjugated diene structure in LDL.

The lipoprotein profiles of III.3 and a normolipidemic subject were substantially different. Interestingly, VLDL was nearly absent, LDL was reduced and HDL was massively increased in the proband's plasma (Fig. 4a). A detailed analysis of all lipoprotein constituents shows a fivefold increase in HDL2 (Table 3) but a lower apoC-III content in the proband's HDL2/HDL3 fractions (Supplementary Fig. 2). In addition, HDL particles from III.3 were enriched in esterified and free cholesterol, and in phospholipid, but depleted in TG and protein, a composition typical of hyperalphalipoproteinemic HDL (Table 4). The profile of several lipid classes in the proband's HDL was altered (Table 5). Although the proband had CKD, his HDL maintained an elevated cholesterol efflux capacity compared with HDL of the normolipidemic control (Fig. 4b), whereas the antioxidative activities of total HDL and small, dense HDL3c were similar between the proband and the normolipidemic control subject (Fig. 4c). The hypotriglyceridemia and hyperalphalipoproteinemia of apoC-III-D25V carriers confer an atheroprotective profile. This was corroborated by clinical data (detailed imaging of coronary angiography, pulse arterial Doppler ultrasound of the lower limbs and supra-aortic trunks, computed tomography (CT) angiography of the aorta and iliac vessels) establishing the absence of atheroma or vascular wall calcification, whereas carotid intima-media thickness was normal in the proband and his sister (III.5). Similarly, there was no record of ischaemic CVD in D25V-patients I.2 or II.1, who died aged 74 and 82 years, respectively.

### Analysis of structure, function and aggregation propensity

**Figure 5:**
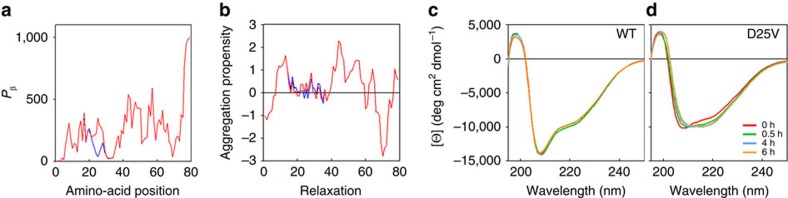
Predicted and experimental beta-aggregation propensity at the site of mutation. (**a**) Protein β-sheet content was calculated using the GOR secondary structure prediction method^53^. (**b**) Aggregation propensity calculated according to the Zyaggregator method^54^. (**c**) Change in secondary structure monitored by far-UV CD during aggregation of both wild-type (WT) and (**d**) D25V apoC-III in PBS, pH 7.4, 37 °C at 1,500 r.p.m. (IKA magnetic stirrer). CD spectra of samples (200 μl) at 0, 0.5, 4, 6 h, respectively, were recorded in 1.0 mm path length quartz cell, buffer subtracted and expressed as mean ellipticity per residue (*θ*).

**Figure 6:**
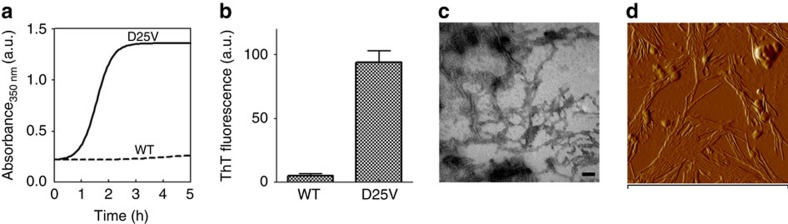
Amyloid fibril formation by D25V apoC-III. (**a**) Increase in turbidity at 350 nm was used to monitor the aggregation of wild-type (WT) and D25V ApoC-III in PBS, pH 7.4, and 1,500 r.p.m. (IKA magnetic stirrer). The curve shown is representative of three replicates. (**b**) Thioflavin T (ThT) fluorescence emission at 480 nm after excitation at 445 nm was performed for each apoC-III isoform at the end of protein aggregation, as shown in Fig. 5a. Bars show the ThT relative intensity fluorescence (arbitrary units, a.u.) of a 10-μl aliquot of protein samples in the presence of 10 μM ThT. Values are shown as mean±s.d. of three independent experiments. (**c**) Negatively stained transmission electron micrograph of D25V apoC-III fibrils generated in physiological conditions. Scale bar, 100 nm. (**d**) Tapping mode atomic force microscopy (AFM) image (amplitude data) of ApoC-III fibrils. AFM measurements were performed in tapping mode with a Multimode Scanning Probe Microscope driven by a Nanoscope IV controller (Digital Instruments, Bruker), using single-beam uncoated silicon cantilevers (type OMCL-AC160TS, Olympus). The drive frequency was between 290 and 320 kHz, the scan rate was between 0.5 and 0.8 Hz. Scan size is 1.2 μm.

Predictive analysis of the secondary structure of the apoC-III polypeptide in its lipid-free state revealed that the D25V mutation enhances both its beta-sheet content and aggregation propensity (Fig. 5a,b). Although wild-type and D25V apoC-III initially showed similar circular dichroic spectra, the mutation appears to cause a transition towards prominent beta-sheet content (Fig. 5c,d) associated with rapid formation of aggregates (Fig. 6a), which were shown to increase the thioflavin T fluorescence^16^, the typical signature of amyloid fibrils (Fig. 6b). Transmission electron microscopy (Fig. 6c) and atomic force microscopy (Fig. 6d) confirmed the presence of fibrillar structures in the D25V apoC-III aggregated material.

**Figure 7:**
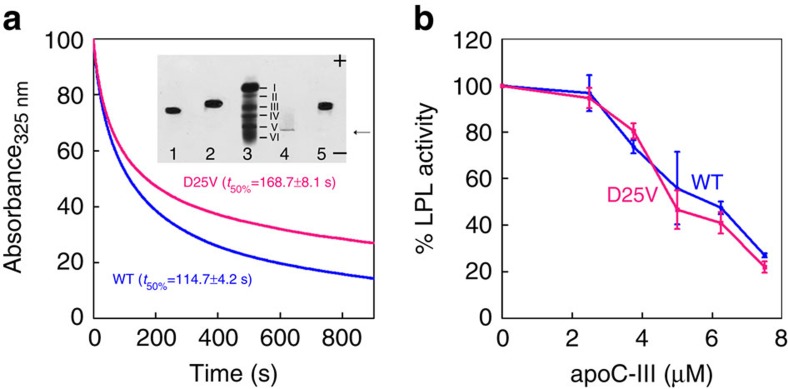
Functional properties of wild-type and D25V apoC-III *in vitro*. (**a**) Lipid binding of ApoC-III was monitored by decrease in turbidity at 325 nm of DMPC vesicles mixed with recombinant wild-type or D25V ApoC-III at a lipid/protein ratio of 2:1 (w/w) over 900 s at 30° C. The binding curves shown are representative of three independent experiments and expressed as percentage of the initial absorbance of the vesicles at 325 nm. The time required to reduce by 50% the initial signal at 325 nm (*t*_50%_) is given as mean±s.d. of three replicates. Inset, native gel electrophoresis shows the shift in mobility of both proteins after formation of the vesicles with DMPC. Lanes: wild-type apoC-III alone (1) and with DMPC (2); marker (3); D25V apoC-III alone (4) and with DMPC (5). Marker for electrophoretic mobility is a fivefold diluted plasma (see Methods for details) in which the bands corresponding to the major plasma protein components are highlighted: albumin (I), α-1 antitrypsin (II), haptoglobin (III), transferrin (IV), fibrinogen (V) and γ-globulin (VI). The arrow indicates the site of deposition of the samples where clearly the apoC-III variant precipitated. Negative and positive gel polarities indicate the direction of the electrophoretic run. (**b**) Inhibition of LPL activity was determined keeping constant the incubation time and measuring the free fatty acid release at different concentrations of the two apoC-III isoforms. Data plotted as mean±s.d. of three independent experiments.

The effect of the mutation on the lipid binding was also carried out using dimyristoylphosphatidylcholine (DMPC) multilamellar vesicles^17^. Analysis of the binding curves based on the decrease in turbidity at 325 nm showed that the time required to reduce by 50% the initial turbidity of the vesicles was 168.7±8.1 s for the variant and 114.7±4.2 s for the wild-type, respectively (Fig. 7a), suggesting that the mutation may cause a less efficient lipid binding similar to other apoC-III variants^17^. At the equilibrium, however, the variant was integrated in the vesicles as shown in the native electrophoresis (inset, Fig. 7a). The native electrophoresis revealed the strong propensity of the variant to self-aggregate in its free form when it aggregated and precipitated at the site of deposition (inset, Fig. 7a). Solubility of D25V apoC-III was, however, rescued when the variant was integrated within the vesicles. We have also investigated the effect of the mutation on the well-known inhibition of LPL activity by apoC-III (refs 3, 18). The D25V apoC-III variant exhibited the same inhibitory activity when compared with the wild-type (Fig. 7b). It is worth noting that another mutation in position 25 (D25N, designed *in silico*), with the change in charge but unmodified hydrophobicity, was as efficient as the wild-type in inhibiting LPL activity^19^.

**Figure 8:**
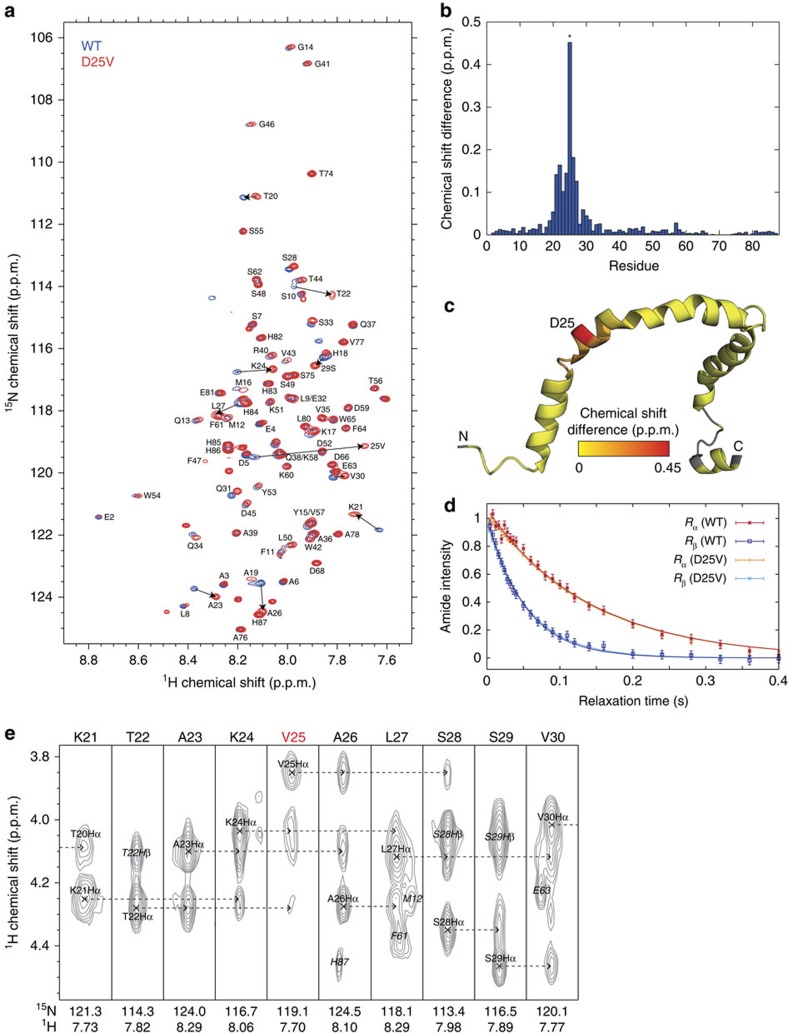
NMR spectroscopy. (**a**) ^1^H,^15^N-Heteronuclear single quantum coherence (HSQC) spectra of wild-type (WT, blue) and D25V (red) apoC-III. Residue assignments of the variant were based on the Gangabadage^9^ assignment of the wild-type protein, using a nuclear overhauser effect spectroscopy (NOESY)-HSQC spectrum (120 ms mixing time) to reassign perturbed residues around the mutation site. Large chemical shift changes from the WT are highlighted with arrows. (**b**) Amide chemical shift differences between WT and D25V apoC-III, calculated as the weighted combination Δ*δ*_NH_=(Δδ_H_^2^+ (Δδ_N_/ 5)^2^)^1/2^. An asterisk (*) highlights the position of residue 25. (**c**) Amide chemical shift differences projected onto the WT NMR structure (pdb2jq3) according to the indicated colour scale. Grey areas indicate unassigned residues or proline. (**d**) TRACT relaxation interference measurements^34^ of WT and D25V apoC-III, determined by integration of the amide envelope and fitting to single exponential decays. Fitted ^15^N relaxation rates±s.e. were 7.2±0.4 s^−1^ (WT) and 7.2±0.2 s^−1^ (D25V) in the α spin state, and 19.3±1.0 s^−1^ (WT) and 18.2±0.4 s^−1^ (D25V) in the β spin state, corresponding to hydrodynamic radii values±s.e. of 20.3±0.6 Å (WT) and 19.6±0.2 Å (D25V). (**e**) ^1^H strips from the NOESY-HSQC spectrum of D25V apoC-III. Dashed arrows highlight sequential NOEs from the H_α_ atom of residue *i* to the H_N_ atom of residue *i*+1/*i*+3, which are diagnostic of α-helical structure.

We finally compared the 3D NMR structure of D25V apoC-III with that of the wild-type counterpart already characterized in the presence of SDS^9^. Two-dimensional ^1^H,^15^N NMR spectra (Fig. 8a) showed small (<0.2 p.p.m., excluding residue 25) amide chemical shift changes in the variant (Fig. 8b,c), which were clustered within approximately one helical turn (3.6 residues) of the mutation site. No chemical shift perturbations were observed elsewhere in the sequence. The hydrodynamic radii of wild-type and D25V apoC-III micellar complexes, recorded by TRACT relaxation interference experiments, were virtually indistinguishable (Fig. 8d). Moreover, there was no difference in the helical conformation of residues 20–30 (Fig. 8e).

## Discussion

Here we report a previously unappreciated, naturally occurring *APOC3* mutation^6^,^7^,^8^, which causes low levels of plasma TG and apoC-III and a favourable lipoprotein profile in a French kindred affected with amyloidosis. Concordance between the *APOC3* mutation and the presence of amyloid among members of this kindred, coupled with identification of D25V apoC-III within amyloid fibrils from numerous affected tissues and *in vitro* demonstration of its amyloidogenicity, establishes that the D25V variant also causes amyloidosis.

The phenotype of this new form of amyloidosis is mainly characterized by onset with sicca syndrome as early as age 20 years, secondary to diffuse salivary gland deposits and progressive renal insufficiency. We showed that these clinical symptoms are caused by amyloid deposition in the respective tissues with progressive and widespread amyloid accumulation within different organs. In this novel form of renal amyloidosis, amyloid deposition is characterized by prominent vascular involvement in all compartments of the kidney and ischaemic glomerular lesions that probably account for low-grade proteinuria and severe hypertension. This clinical renal presentation contrasts those described for Leu75Pro apoA1 amyloidosis^20^ and A-alpha chain amyloidosis^21^, which typically manifests as a chronic tubulo-interstitial nephritis with amyloid deposits restricted to the inner medulla, or as proteinuria/nephrotic syndrome because of massive glomerular amyloid deposition, respectively, ApoC-III should be added to the list of protein variants associated with hereditary renal amyloidosis^22^, along with apoA-I (ref. 10, apoAII (ref. 11), lysozyme^23^, fibrinogen Aα-chain^21^ and gelsolin^24^. The diagnosis of apoC-III amyloidosis may be suggested by hypotriglyceridemia, present in all amyloidotic patients from this kindred, a biological marker that is usually absent in patients with other forms of hereditary renal amyloidosis.

Our *in vitro* studies clearly show that, despite the remarkable differences in folding dynamics and kinetics of aggregation between wild-type and D25V apoC-III in their lipid-free state, their conformation and colloidal stability are similar in the lipid-bound state. Wild-type apoC-III was recently reported to self-aggregate into polymeric ordered structures with a peculiar triangular geometry and a Möbius strip conformation after 3 days of shaking in physiological buffer. Although these structures do not resemble the genuine amyloid fibrils that we obtained under physiological conditions with the D25V variant, it is worth noting that aggregation of lipid-free wild-type apoC-III was preceded by a gradual structural transition that resembles, in circular dichroism (CD) spectroscopy, the conformation rapidly adopted by the D25V variant. There is no evidence suggesting *in vivo* amyloidogenicity of wild-type apoC-III and demonstration of aggregation *in vitro* does not necessarily imply amyloidogenicity *in vivo.* Importantly, the absence of wild-type apoC-III within the *ex vivo* amyloid fibrils from three patients in this kindred suggests that the wild-type protein does not contribute to amyloid deposition even in the presence of abundant D25V fibrillar seeds, analogous to what we have recently observed in patients with familial β_2_-microglobulin amyloidosis^14^.

We hypothesize that the preferential incorporation of D25V apoC-III into amyloid fibrils is likely the main cause of the imbalance in the ratio of wild-type/D25V apoC-III observed within HDL particles. It will be worth investigating further in appropriate cellular and/or animal models whether the secretion of the variant may be partially impaired by the intracellular quality control, similar to what we previously described for some of the amyloidogenic variants of apoA-I (ref. 25), in which we found an imbalance in the wild type/variant ratio.

D25V-carriers displayed a reduction in plasma and HDL apoCIII concentration, hypotriglyceridemia associated with a dramatic decrease in the number of VLDL particles, and a concomitant massive increase in the larger HDL2 fraction. We propose that these are indirect effects of the D25V variant because of its very low concentration in plasma (and HDL fractions) because of its preferential association with amyloid fibrils. The lower plasma apoC-III concentration in D25V-carriers probably results in lack of inhibition of LPL and LDL-R. As a consequence, VLDL-TG hydrolysis proceeds at high rate and is followed by rapid uptake of VLDL-remnants (or intermediate density lipoprotein (IDL) in Fig. 4b), provoking the drastic decreases in VLDL and IDL of D25V-carriers (Fig. 4a,b). It has been well established that surface lipids of VLDL, which are liberated after LPL-mediated VLDL-TG hydrolysis, are transferred to HDL by phospholipid transfer protein, resulting in an increase in HDL size and particle number, as confirmed in mice deficient either in LPL^26^ or in phospholipid transfer protein^27^ and which lack HDL. Importantly, subjects heterozygous for other loss-of-function *APOC3* mutations (K58E^28^ and R19X^5^,^6^) also present with hypotriglyceridemia and increased plasma HDL.

The favourable lipid/lipoprotein profile of D25V-carriers is remarkable in the context of CKD and/or ESRD, pathological conditions predisposing to premature atherosclerosis and major ischaemic CVD events and commonly associated with an unfavourable lipid profile, an elevated plasma apoC-III concentration and dysfunctional HDL carrying increased amounts of apoCIII, serum amyloid A1 and lipoprotein-associated phospholipase A2 (refs 29, 30, 31). Hypertriglyceridemia, often due to accumulation of VLDL remnants/IDL in the circulation, is an independent CVD risk factor^32^, and low plasma HDL is a second independent CVD risk factor^33^. D25V-carriers displaying hypotriglyceridemia and very high HDL2 are obviously lacking in CVD risk factors. Moreover, their HDL is functional, as evidenced by their normal antioxidant activity and cholesterol efflux capacity possibly related to their enrichment in sphingomyelin (SM). Furthermore, the lower apoC-III content of HDL may contribute to atheroprotection as HDL particles without apoC-III reduce monocyte adhesion to vascular endothelial cells, whereas apoC-III-rich HDL do not, suggesting that apoC-III may have additional proatherogenic effects on vascular endothelial cells^34^. Thus, the pathologic amyloidogenic properties of D25V apoC-III unexpectedly confer a favourable cardioprotective lipoprotein profile indirectly, most probably through lowering of plasma apoC-III concentration, extending the evidence for an important antiatherogenic role of *APOC3* deficiency in ESRD patients.

As reducing the amyloid fibril precursor protein concentration is known to slow amyloid formation and improve prognosis among patients with a variety of systemic amyloidoses, and as PPARα agonists (fibrates) significantly reduce hepatic *APOC3* transcription^35^,^36^, we postulate that fibrate therapy may have therapeutic potential for treatment of this new form of amyloidosis. Because the safety and side-effect profiles of fibrates are well known, it might be reasonable to prescribe fibrates to the affected individuals from this kindred in an attempt to slow down amyloid formation. If successful, this therapy could represent an alternative to the gene silencing approach currently under evaluation in other systemic amyloidoses^37^.

## Methods

### Patients

The study was carried out in accordance with the Declaration of Helsinki, and was approved by the University of Marseille Institutional Review Board. All authors vouch for the completeness and accuracy of the analyses and results. Oral and written informed consents were obtained from all participants, including the transfer of biopsy material outside of France.

### Genetic analysis

Genomic DNAs were extracted from peripheral blood samples, using the QIAamp DNA mini kit (Qiagen). Coding and consensus splice site regions of *APOA1*, *APOA2*, *TTR*, *B2M*, *FGA*, *LYZ*, *GSN*, *SAA, APOE, APOC3*, *APOC2*, *APOC1*, *APOC4*, *APOA4* and *APOA5* genes were amplified by PCR (Ampli*Taq* Gold; ABI, or Platinium *Taq*; Invitrogen), according to the manufacturer's instructions, in a DNA thermal cycler 9700 (Perkin-Elmer). The primer sequences for *APOC3* amplification are listed in Supplementary Table 2. The resulting PCR fragments were purified by QIAquick PCR Purification kit (Qiagen), and sequenced using the Big Dye terminator cycle sequencing kit (DNA sequencing kit; Applied Biosystems) on an automatic genetic analyser (ABI PRISM 3100 genetic analyzer; Applied Biosystems)^14^. *APOC3* polymorphims at positions −455 (rs 2854116), and −482 (rs2854117) in the promoter region were genotyped by direct sequencing. Mutation nomenclature was based on the *APOC3* transcript reference (NCBI RefSeqcDNA accession number NM_000040.1), and nucleotides were numbered according to the cDNA with +1 corresponding to the A of ATG translation initiation codon according to the Human Genome Variation Society guidelines, http://www.hgvs.org/mutnom//. Therefore, the apoC-III variant is described as p.Asp45Val (with the signal peptide), whereas the mature protein is designed as D25V.

### Histology and immunohistochemistry

Amyloid was detected by Congo red staining of formalin-fixed wax-embedded biopsy sections^14^ (kidney, salivary glands, heart, bowel, fat and bronchia from III.3; kidney, salivary glands, skin, heart, liver from III.5; salivary glands, kidney, skin, kidney, bowel from II.3; salivary glands from IV.1, IV.3, and IV.4). Amyloid typing was performed by indirect immunofluorescence and by immunoelectron microscopy^14^. For indirect immunofluorescence, 3 μm cryostat sections of frozen tissue were air dried, incubated 1 h with primary goat anti-human ApoC3 polyclonal antibody (Abnova, 50 μg ml^−1^ in PBS), washed with PBS and stained with fluorescent isothiocyanate-conjugated donkey anti-goat antibody IgG, F(ab,)2 (Santa Cruz, 20 μg ml^−1^ in PBS). For the immunogold labelling procedure, thin sections (80 nm) were cut from the routine (glutaraldehyde-fixed, osmicated, Araldite embedded) electron microscopic blocks, collected on nickel grids. Grids were floated on one drop of 5% sodium metaperiodate (Sigma Chemical Co) in distilled water for 30 min. Sections were washed with distilled water three times and then saturated for 1 h with 3% BSA in PBS in order to minimize nonspecific labelling. The grids were then incubated overnight at 4 °C with primary goat anti-human ApoC3 polyclonal antibody (Abnova, 100 μg ml^−1^in PBS).

Sections were washed six times with PBS, incubated for 1 h with a rabbit polyclonal biotin-conjugated anti-goat IgG secondary antibody (Clinisciences, 100 μg ml^−1^ in PBS) and 1 h with gold-labelled strepatavidin (Zymed Laboratories, 6 μg ml^−1^ in PBS). Immunolabelled sections were contrasted using uranyl acetate and observed under a JEOL 1010 transmission electron microscope (Jeol Ltd). In all cases, parallel control negative sections consisting of the absence of the primary apoC-III antibody were performed, demonstrating the specificity of the apoC-III antibody.

### LMD and tandem MS-based proteomics

Congo red-stained sections from salivary glands (IV.3), heart and digestive tract (III.3) were examined for the presence of amyloid under fluorescence (Leica, B/G/R filter cube). Positive areas were microdissected into 0.5 ml microcentrifuge tube caps containing 10 mM Tris, 1 mM EDTA, 0.002% Zwittergent 3–16 (Calbiochem) using a Leica DM6000B Microdissection System (Leica). For each case, —two to four different microdissections (60,000 μm^2^ each) were collected. Collected tissues were heated at 98 °C for 90 min with occasional vortexing. Following 60 min of sonication in a waterbath, samples were digested overnight at 37 °C with 1.5 μl of 1 μg ml^−1^ trypsin (Promega)^15^. The trypsin generated digests were reduced with dithiothreitol and separated by nanoflow liquid chromatography-electrospray tandem mass spectrometry using a ThermoFinnigan LTQ Orbitrap Hybrid Mass Spectrometer (Thermo Electron) coupled to an Eksigent nanoLC-2D HPLC system (Eksigent). A 0.25-μ1 trap (Optimize Technologies) packed with Michrom Magic C-8 was plumbed into a 10-port valve. A 75 μm × ∼15 cm C-18 column was utilized for the separation utilizing an organic gradient from 6 to 86% in 55 min at 400 nl min^−1^. The Thermo-Fisher MS/MS raw data files were searched using three different algorithms (Mascot, Sequest and X!Tandem) and the results assigned peptide and protein probability scores. The results were then combined and displayed using Scaffold (Proteome Software). All searches were conducted with variable modifications and restricted to full trypsin-generated peptides allowing for two missed cleavages. Peptide mass search tolerances were set to 10 p.p.m. and fragment mass tolerance to 1.00 Da. The human SwissProt database (24 July 2007) was complemented with frequently observed contaminants (porcine trypsin and human keratins). Protein identifications below the 90% confidence level and those with single peptide identification were not considered

### Lipid and lipoprotein analysis

Plasma levels of TGs and HDL-cholesterol were measured in the non-fasting state using standard hospital biochemical assays. Plasma apoC-III concentrations were measured by electro-immunodiffusion. Lipoprotein classes were prepared by sequential ultracentrifugation of sera from the proband III-3 and a control subject run in parallel. Sequential ultracentrifugations were performed at 100,000*g* and 10 °C to isolate VLDL, IDL and LDL (for 18 h, at densities 1.006, 1.020 and 1.063 g ml^−1^, respectively) and HDL (for 40 h, at density 1.21 g ml^−1^) in an OPTIMA XL 100 K Beckman ultracentrifuge^38^. After extensive dialysis of all lipoproteins against PBS containing/0.3 mM EDTA, their protein contents were measured with the Bio-Rad DC Protein assay kit II (Bio-Rad), and their TG, total cholesterol and phospholipid with commercial kits (DiaSys). HDL apolipoproteins were analysed by 15% SDS–PAGE under non-reducing conditions to distinguish the apoA-II homodimer (17 kDa) from apo Cs^38^, and stained with Coomassie Brilliant Blue G 250. Purified human apoA-I and apoA-II, as major HDL apolipoproteins, were run as standards (4 μg per lane) in the gel. For apoC-III detection, proteins were transferred to a nitrocellulose membrane and immunoblotted with rabbit anti-human apoC-III antibody (1 μg ml^−1^ in PBS, Abnova) and secondary ECL anti-rabbit IgG-HRP-Linked specific whole antibody (5,000-fold diluted in PBS, GE Healthcare). Purified human apoC-III (4 μg per lane) was added as standard in the western blotting. Protein bands were visualized with ECL Western Blotting Detection Reagent (GE Healthcare). Standard human apoA-I, apoA-II and apoC-III were purified from delipidated HDL by preparative isoelectric focusing with a pH gradient 4–6.

### Fractionation of HDL subpopulations

HDL subpopulations were isolated from plasma (3 ml) by single step, isopycnic non-denaturing density gradient ultracentrifugation in a Beckman SW41 Ti rotor at 40,000 r.p.m. for 44 h in a Beckman XL70 ultracentrifuge at 15 °C by a slight modification of the method of Chapman *et al.*^39^,^40^. After centrifugation, each gradient was fractionated in predefined volumes from the meniscus downwards with an Eppendorf precision pipette into 11 fractions corresponding to very-low-density lipoprotein and intermediate-density lipoprotein (VLDL+IDL; 800 μl, *d*<1.019 g ml^−1^), LDL (5 subfractions, 800 μl each, LDL1, *d*=1.019–1.023 g ml^−1^; LDL2, *d*=1.023–1.029 g ml^−1^; LDL3, *d*=1.029–1.039 g ml^−1^; LDL4, *d*=1.039–1.050 g ml^−1^ and LDL5, *d*=1.050–1.063 g ml^−1^) and HDL. Five major HDL subclasses were isolated, that is, large, light HDL2b (1,200 μl, *d*=1.063–1.087 g ml^−1^) and HDL2a (800 μl, *d*=1.088–1.110 g ml^−1^), and small, dense HDL3a (*d*=1.110–1.129 g ml^−1^), HDL3b (800 μl, *d*=1.129–1.154 g ml^−1^) and HDL3c (800 μl, *d*=1.154–1.170 g ml^−1^). The validity and reproducibility of this density gradient procedure, which facilitates preparative fractionation of HDL particle subspecies in a non-denaturated, native state, have been extensively documented^39^,^40^,^41^. All HDL subclasses isolated by this procedure are essentially albumin free (<1% of total protein, that is, <0.05 mg dl^−1^)^42^. Lipoproteins were extensively dialysed (3 cycles of at least 8 h) against PBS pH 7.4 at 4 °C in the dark, stored at 4 °C and used within 8 days.

### Phosphosphingolipidome analysis

For the phosphosphingolipidome analysis, HDL subpopulations (30 μg phospholipid mass determined using a commercially available assay) were added to 4 ml of cold CHCl_3_/acidified CH_3_OH (5:2 (v/v)) containing seven internal lipid standards (4 μg of phosphatidylcholine d9 32:0, 100 ng of phosphatidylinositol (PI) 25:0, 80 ng of phosphatidylethanolamine, 25:0, 80 ng of phosphatidic acid 25:0, 40 ng of phosphatidylserine 25:0, 20 ng, of phosphatidylglycerol 25:0 and 20 ng of ceramide 17:0; Avanti Polar Lipids)^43^. A blank and a control sample were extracted in parallel with each batch to ensure for quality control. K_4_EDTA (200 mM) solution was added (1:5 (v/v)) and the mixture was vortexed and centrifuged. The organic phase was dried under nitrogen and lipids were reconstituted into isopropanol/hexane/water (10:5:2 (v/v)), transferred into LC/MS vials, dried under nitrogen and resuspended in isopropanol/hexane/water (10:5:2 (v/v)). Seven principal phospholipid (PL) subclasses (phosphatidylcholine, lysophosphatidylcholine, phosphatidylethanolamine, PI, phosphatidylglycerol, phosphatidylserine and phosphatidic acid) and two principal sphingolipid (SL) subclasses, SM and ceramide, which together comprise >160 individual molecular lipid species and account for >95% of total plasma PL and SM^44^ were assayed by LC/MS/MS. Lipids were quantified using a QTrap 4000 mass spectrometer (AB Sciex), an LC20AD HPLC system, and the Analyst 1.5 data acquisition system (AB Sciex). Quantification of PLs and SLs was performed in positive-ion mode, except for PI species that were detected in negative-ion mode. Sample (4 μl) was injected onto a Symmetry Shield RP8 3.5 μm, 2.1 × 50 mm^2^ reverse phase column (Waters Corporation) using a gradient from 85:15 to 91:9 (v/v) methanol/water containing 5 mM ammonium formate and 0.1% formic acid at a flow rate of 0.1 ml min^−1^ for 30 min. Lipid species were detected using multiple reaction monitoring reflecting the head-group fragmentation of each lipid class and quantified using calibration curves specific for the nine individual lipid classes with up to 12 component fatty acid moieties; 23 calibration curves were generated in non-diluted and tenfold diluted matrices to correct for matrix-induced ion suppression effects. More abundant lipid species that displayed a nonlinear response in non-diluted extracts were quantified from a 10- or 100-fold diluted sample. An in-house developed script was used to compile data from the three successive injections.

### Cellular cholesterol efflux capacity of HDL

The cholesterol efflux capacity of HDL was characterized in a human THP-1 monocytic cell system (ATCC)^45^. In this assay, HDL particles were compared on the basis of their PL concentrations because PL was shown to represent the key component determining cholesterol efflux capacity of HDL^46^. Briefly, THP-1 monocytes were cultured on 24-well tissue culture plates, grown in RPMI-1640 media with 10% FBS and differentiated into macrophage-like cells with 50 ng ml^−1^ phorbol 12-myristate 13-acetate for 48 h and 37 °C. The cells were washed and loaded for 24 h with [3H]cholesterol-labelled acetylated LDL (1 μCi ml^−1^) in serum-free RPMI-1640 culture medium supplemented with 50 mM glucose, 2 mM glutamine, 0.2% BSA, 100 μg ml^−1^ penicillin and 100 μg ml^−1^ streptomycin (further abbreviated as RGGB) to allow equilibration of cellular cholesterol pools. The labelling medium was removed and human macrophages were then equilibrated in RGGB for an additional 16–24 h period. Cellular cholesterol efflux to 15 μg ml^−1^ HDL-PL was assayed in 300 μl serum-free medium for a 4-h chase period. Finally, culture media were harvested and cleared of cellular debris by brief centrifugation. Cell radioactivity was determined by extraction in hexane-isopropanol (3:2), evaporation of the solvent under nitrogen and liquid scintillation counting (Wallac Trilux 1450 Microbeta, Perkin Elmer). The percentage of cholesterol efflux was calculated as (medium cpm)/ (medium cpm+cell cpm) × 100%. Specific cholesterol efflux was determined by subtracting nonspecific cholesterol efflux occurring in the absence of cholesterol acceptors.

### Antioxidative activity of HDL

Antioxidative activity of HDL (final concentration of each, 10 mg total mass per dl) was assessed at a physiological HDL to LDL ratio towards reference LDL (*d*=1.019–1.063 g ml^−1^; final concentration, 10 mg dl^−1^ TC) isolated from one healthy normolipidemic control subject^42^,^47^,^48^. In this assay, HDL particles were compared on the basis of their total mass concentrations because both protein and lipid components were shown to contribute to the capacity of HDL to inhibit LDL oxidation^43^,^49^,^50^. HDL subfractions were added to LDL directly before oxidation. Lipoprotein oxidation was induced by an azo-initiator 2,2′-azo-bis-(2-amidinopropane) hydrochloride (final concentration 1 mmol l^−1^) as model of mild oxidation induced by free radicals in the arterial intima^42^,^47^,^48^. Serum was used as a source of HDL for this assay to ensure intact paraoxonase activity, which is inhibited by EDTA. Thereby, this assay employs mild oxidative conditions and integrates the antioxidative activities of several HDL components, that is, apoA-I, antioxidative enzymes and lipophilic low-molecular-weight antioxidants^43^,^49^,^50^. Accumulation of conjugated dienes was measured as the increment in absorbance at 234 nm. Absorbance kinetics values were corrected for the absorbance of 2,2′-azo-bis-(2-amidinopropane) hydrochloride itself run in parallel as a blank. Three consecutive phases were identified, the lag, the propagation and the decomposition phases for each curve and duration of the propagation rate, oxidation rate in the propagation phase and amount of dienes formed at the end of the propagation phase (maximal concentration of dienes) were calculated as markers of antioxidative activity of HDL^42^,^47^,^48^.

### MS analysis of apoC-III isoforms from HDL

Twenty micrograms of HDL fraction was dissolved and acidified in 20 μl of 0.1% trifluoroacetic acid for 15 min with agitation. The protein was desalted using ZipTips C18 (Millipore) following the manufacturer's instructions and eluted with 20 μl of saturated matrix solution of sinapinic acid in 50% acetonitrile and 0.5% (v/v) trifluoroacetic acid. The mass spectrometric measurements were performed using a MALDI TOF/TOF 4800 Proteomics Analyzer mass spectrometer^51^. The MS spectra from *m*/*z* 8,000 to 10,000 Da were acquired both in reflectron and in linear positive ion modes using 1,000 laser shots. For mass spectrometric identification of ApoC-III in HDL2/3 using LC-MS/MS, 25 μg of HDL fraction was dissolved into 500 mM triethylammonium bicarbonate, 1% SDS. Proteins were reduced with 10 mM dithiothreitol, alkylated with 20 mM iodoacetamide and trypsin digested overnight. The resulting peptides were cleaned up using OMIX C18 100 μl pipette tips (Agilent), and lyophilized before being reconstituted for the LC-MS/MS analysis. The peptides were separated using an Eksigent Ultra nano-LC HPLC system coupled with an AB Sciex Triple TOF 5600 mass spectrometer. The LC separations were performed using a Discovery Bio Wide Pore HPLC column (C18, 3 μm, 100 × 5 mm^2^). The mobile phases used were 0.1% formic acid in water (A) and 100% acetonitrile with 0.1% formic acid (B). The gradient elution steps were performed with a flow rate of 5 μl min^−1^ as follows: 0–40% B for 106 min, 40–80% for 5 min and then 80% B for an additional 5 min. All data were acquired using Analyst software (AB Sciex) in the data-dependent mode. Peptide profiling was performed using a mass range of 350–1,600 Da, followed by a MS/MS product ion scan from 100 to 1,500 Da. For each survey MS1 scan (accumulation time of 250 ms), MS/MS spectra (accumulation time of 75 ms per MS/MS) were obtained for the 30 most abundant precursor ions. The protein identification was performed with the ProteinPilot Software 4.5.0 (AB Sciex). The MS/MS spectra obtained were searched against the UniProt database (release 2014_03). The search parameters for tryptic cleavage and accuracy are built-in functions of the software. Other data analysis parameters were as follows: sample type: identification; Cys-alkylation: iodoacetamide; digestion: trypsin; instrument: TripleTOF 5600; special factor: biological modifications; species: Homo sapiens; search effort: thorough ID. Proteins comprising one or more peptides with a high confidence score (95%) and a low false discovery rate (estimated local false discovery rate of 1%) were considered positively identified.

### Radiolabelled SAP scintigraphy

Patients III.3 and IV.3 underwent ^123^I-labelled SAP scintigraphy^52^ (http://www.amyloidosis.org.uk/diagnosis/sap-scintigraphy-3/). Purified human SAP was labelled with ^123^I using the N-bromosuccinimide method. Each patient received ∼100 μg SAP bearing 200 MBq of ^123^I, corresponding to an effective dose equivalent of ∼3 mSv. Thyroid uptake was blocked by oral ingestion of potassium iodide for 2 days before and following isotope injection. Anterior and posterior whole-body scintigraphy was performed 24 h after isotope administration using a GE Healthcare-Discovery gamma-camera.

### Expression and purification of recombinant ApoC-III

ApoC-III was expressed from a pET23b vector containing the full-length cDNA for human ApoC-III, including the sequence encoding a C-terminal His6-tag preceded by Leu and Glu additional residues^17^. pET23b/D25V was generated by using a QuikChange site-directed mutagenesis kit (Stratagene). In a 50 ml reaction, 50 ng of plasmid DNA pET23b/*ApoC-III* was used as template and two synthetic oligonucleotide primers, the forward primer (5′-CCAAGACCGCCAAGGtTGCACTGAGCAGCG-3′) and the reverse primer (5′-CGCTGCTCAGTGCAaCCTTGGCGGTCTTGG-3′) with the A-to-T base change complementary to opposite strands of the vector, were extended by *Pfu* Turbo DNA polymerase (Stratagene). For PCR, there was an initial cycle of 95 °C for 30 s, followed by 12 cycles of 95 °C for 30 s, 55 °C for 1 min and 68 °C for 2 min. After *Dpn*I digestion of the parental deoxyadenosine methylase-methylated template, the synthesized mutated DNA was transformed into *E. coli* XL1Blue supercompetent cells. Candidate clones were screened by sequencing the *ApoC3* insert on an ABI 377 prism DNA sequencer.

### Prediction of beta aggregation propensity

The Garnier-Osguthorpe-Robson (GOR) method version IV (ref. 53) was used to predict the beta propensity at the mutation site. The aggregation propensities of wild-type and D25V ApoC-III were predicted using the Zyggregator method^54^.

### Fibrillogenesis

Fibrillogenesis experiments were performed in standard quartz cells stirred at 1,500 r.p.m. (IKA magnetic stirrer) at 37 °C using 100 μM ApoC-III isoforms in PBS, pH 7.4. Aggregation was carried out without seeds of preformed fibrils and the increase in turbidity at 350 nm was monitored. Thioflavin T fluorescence emission^16^ was measured at the end-point aggregation. Transmission electron microscopy analysis was performed using protein samples stained with 2% (w/v) uranyl acetate using a CM120 microscope at 80 keV. Atomic force microscopy analysis was carried out on 10 μl of fibrillar sample incubated on a freshly cleaved mica substrate for 5 min, then rinsed and dried. Conformational modifications during aggregation of apoC-III were monitored by far-UV CD.

### Lipid-binding properties of apoC-III

Association of recombinant wild-type and D25V apoC-III to DMPC multilamellar vesicles^55^, at a lipid-to-protein ratio of 2:1 (w/w), was followed by monitoring the decrease in turbidity of DMPC liposomes at 325 nm as a function of time at 30 °C (ref. 17) in 0.01 M Tris-HCl buffer pH 8.0 containing 150 mM NaCl, 8.5% KBr, 0.01% NaN_3_ and 0.01% EDTA using a 1 cm path length quartz cell in a Jasco V-650 spectrophotometer equipped with a Peltier temperature controller. The lipid/protein association was expressed as percentage of the turbidity of DMPC vesicles in the absence of apoC-III; the time required to reduce the initial absorbance at 325 nm by 50% was determined from the curves to compare the lipid-binding properties of the two apoC-III isoforms. Electrophoretic mobility of wild-type and D25V apoC-III with and without DMPC was analysed using 1% medium electroendosmosis agarose gel in 70 mM barbiturate-sodium barbiturate buffer, pH 8.6 containing 2 mM calcium lactate. Samples (3 μl) were applied to the gel including fivefold diluted human plasma in 2% bromophenol blue solution used as marker for protein electrophoretic mobility. Electrophoresis was carried out in a 10 °C water-thermostated chamber at 20 V cm^−1^. Gel was then fixed for 15 min in a 15% acetic acid solution containing 1% picric acid, dried and stained with Coomassie Blue.

### LPL inhibition

Bovine LPL catalytic activity, in the presence of apoC-III was measured using an emulsion with the same composition as Intralipid 10% (Fresenius-Kabi, Sweden) containing [3H]triolein substrate^56^. The incubation medium contained 2% (v/v) of the emulsion, 0.1 M NaCl, 60 mg ml^−1^ BSA, 0.15 M Tris-HCl, pH 8.5, and 16.7 U ml^−1^ heparin. The medium was pre-incubated with or without each apoC-III sample for at least 10 min before addition of bovine LPL (0.25 μg ml^−1^) in a final 200 μl mixture. After 30 min incubation under agitation at 25 °C, the reaction was stopped by adding 2 ml of isopropanol, heptane and 1 M H_2_SO_4_ (40:48:3:1) and 0.5 ml of water. Free fatty acids were extracted using sequential centrifugations in glass tubes for 3 min at 2,580*g* to separate each mixture in two phases. After the first centrifugation, 800 μl of the upper phase was transferred to other vials containing alkaline ethanol and then added with 3 ml of heptane before mixing and centrifuging again. After removal of the upper phase, 3 ml of heptane were mixed to the lower phase, which was then centrifuged as above. Supernatant was removed and 800 μl of the lower phase transferred to a vial containing 2 ml of scintillation liquid to count radioactivity and quantify the [3H] free fatty acids (nmol ml min^−1^). The LPL activity was then expressed as percentage of the enzyme activity in the absence of apoC-III.

### NMR spectroscopy

NMR spectra were acquired for 0.5 mM samples of wild-type and D25V apoC-III (10 mM sodium acetate (pH 5.0), 180 mM SDS, 10% D_2_O) at 315 K, using a 700-MHz BrukerAvance III spectrometer equipped with a TXI cryoprobe, and processed with nmrPipe and CCPN Analysis.

## Additional information

**How to cite this article:** Valleix, S. *et al.* D25V apolipoprotein C-III variant causes dominant hereditary systemic amyloidosis and confers cardiovascular protective lipoprotein profile. *Nat. Commun.* 7:10353 doi: 10.1038/ncomms10353 (2016).

## Supplementary Material

Supplementary Information Supplementary Figures 1-2 and Supplementary Tables 1-2

## Figures and Tables

**Table 1:** Plasma TG, HDL-C and APOC-III levels.

	**II.3**	**III.1**	**III.3**	**III.4**	**III.5**	**IV.1**	**IV.2**	**IV.3**	**IV.4**
TG (g l^−1^)	0.30	1.80	0.31	1.38	0.44	0.34	0.86	0.35	0.50
HDL-C (g l^−1^)	ND	0.35	0.71	0.57	0.79	0.59	ND	0.69	0.83
ApoC-III (mg l^−1^)	ND	54.0	17.7	ND	ND	ND	44.2	18.8	34.4

apoC-III, apolipoprotein C-III; HDL-C, high-density lipoprotein-cholesterol; ND, not determined; TG, triglyceride.

II.3, III.1, III.3, III.4, III.5, IV.1, IV.2, IV.3, IV.4—refers to individuals in Fig. 1a.

**Table 2:** Relative quantification of apoC-III isoforms in D25V-carriers.

	**HDL2**				**HDL3**			
	**III.4**	**III.3**	**IV.3**	**IV.4**	**III.4**	**III.3**	**IV.3**	**IV.4**
	***D25V−/−***	***D25V+/−***	***D25V+/−***	***D25V+/−***	***D25V−/−***	***D25V+/−***	***D25V+/−***	***D25V+/−***
ApoC-III_0_	8.2	11.8	3.8	3.4	11.2	17.8	5.9	5.9
ApoC-III_1_	60.3	44.1	46.4	51.4	62.7	45.9	53.7	53.7
ApoC-III_2_	31.5	44.1	49.8	45.2	26.1	36.3	40.4	40.4
Ratio WT/D25V Apo-CIII_1_	—	2.4	2.1	2.4	—	1.6	1.7	2.0
Ratio WT/D25V Apo-CIII_2_	—	1.9	1.6	1.7	—	2.1	1.6	1.8

apoC-III, apolipoprotein C-III; WT, wild type.

Relative quantification of each apoC-III isoform in three D25V-carriers (III.3, IV.3 and IV.4) in comparison with a healthy non-D25V-carrier (III.4). The ratio of apoC-III isoforms is modified in the D25V-carriers and less D25V variant than wild-type apoC-III is present in HDL2/HDL3.

**Table 3:** Lipoprotein composition.

	**Protein**		**TG**		**TC**		**PL**		**Total (mg dl**^−1^)
	**(mg dl**^**−1**^)	**(%)**	(**mg dl**^**−1**^)	**(%)**	**(mg dl**^**−1**^)	**(%)**	**(mg dl**^**−1**^)	**(%)**	
*Control*									
VLDL	12.55	(9.3)	87.80	(65.0)	8.55	(6.3)	26.26	(19.4)	135.16
IDL	4.56	(20.6)	8.45	(38.1)	2.99	(13.5)	6.16	(27.8)	22.16
LDL	82.90	(35.2)	13.72	(7.2)	39.13	(20.4)	71.47	(37.3)	207.22
HDL2	22.79	(47.4)	2.86	(5.9)	8.19	(17.0)	14.26	(29.6)	48.09
HDL3	93.15	(63.1)	4.63	(3.1)	13.23	(9.0)	36.70	(24.9)	147.71
									
*Proband (III.3)*									
VLDL	3.55	(11.4)	20.39	(65.7)	1.60	(5.1)	5.51	(17.8)	31.04
IDL	0.42	(4.9)	3.87	(45.9)	1.32	(15.7)	2.82	(33.4)	8.43
LDL	45.80	(31.7)	11.36	(7.9)	30.98	(21.4)	56.53	(39.1)	144.67
HDL2	117.22	(47.5)	5.02	(2.0)	40.19	(16.3)	84.63	(34.3)	247.06
HDL3	62.97	(56.6)	2.79	(2.5)	12.72	(11.4)	32.79	(29.5)	111.27

HDL, high-density lipoprotein; IDL, intermediate density lipoprotein; LDL, low-density lipoprotein; PL, phospholipid; TC, total cholesterol; TG, triglyceride; VLDL, very-low-density lipoprotein.

Lipoproteins of the proband and of a control subject were isolated in parallel by sequential ultracentrifugation of serum (see Methods for details). All lipoprotein components were expressed in mg dl^−1^; in parentheses are the percentages of components in each lipoprotein class.

Total is the sum of all constituents in each lipoprotein class.

**Table 4:** Chemical composition (WT%) of HDL subpopulations.

	**Subjects**	**HDL2b**	**HDL2a**	**HDL3a**	**HDL3b**	**HDL3c**
CE (WT%)	Proband	29.8	21.1	22.2	27.0	20.1
	Control	26.0	23.3	21.6	13.1	12.6
FC (WT%)	Proband	7.5	3.1	3.8	6.2	4.2
	Control	6.5	4.1	3.3	4.5	1.7
PL (WT%)	Proband	31.9	24.4	26.7	31.8	26.6
	Control	32.0	25.8	25.6	24.9	16.9
TG (WT%)	Proband	2.2	1.4	1.4	2.1	1.9
	Control	3.1	2.5	1.9	2.0	1.0
Total protein (WT%)	Proband	28.6	49.9	46.0	32.9	47.1
	Control	32.4	44.4	47.6	55.4	67.9

CE, cholesteryl ester; FC, free cholesterol; HDL, high-density lipoprotein; PL, phospholipid; TG, triglyceride.

Data are shown as WT% of total HDL mass. The proband corresponds to individual III.3, and the control to one healthy normolipidemic subject.

**Table 5:** Phospholipid and sphingolipid composition (WT%) of total HDL.

	**PC**	**LPC**	**SM**	**Cer**	**PE**	**PI**	**PS**	**PG**	**PA**
Proband	78.6	0.59	18.1	0.211	1.2	1.1	0.030	0.008	0.013
Control	83.0	0.44	12.9	0.160	1.8	1.7	0.055	0.006	0.006

Cer, ceramide; LPC, lysophosphatidylcholine; PA, phosphatidic acid; PC, phosphatidylcholine; PE, phosphatidylethanolamine; PG, phosphatidylglycerol; PI, phosphatidylinositol; PS, phosphatidylserine; SM, sphingomyelin.

Data are shown as WT% of total phospholipids+sphingolipids. The proband corresponds to individual III.3, and the control to one healthy normolipidemic subject.

## References

[b1] Havel R. J., Kane J. P. & Kashyap M. L. Interchange of apolipoproteins between chylomicrons and high density lipoproteins during alimentary lipemia in man. J. Clin. Invest. 52, 32–38 (1973) . 4345202 10.1172/JCI107171 PMC302224

[b2] Ooi E. M., Barrett P. H., Chan D. C. & Watts G. F. Apolipoprotein C-III: understanding an emerging cardiovascular risk factor. Clin. Sci. 114, 611–624 (2008) . 18399797 10.1042/CS20070308

[b3] Wang C. S., McConathy W. J., Kloer H. U. & Alaupovic P. Modulation of lipoprotein lipase activity by apolipoproteins. Effect of apolipoprotein C-III. J. Clin. Invest. 75, 384–390 (1985) . 3973011 10.1172/JCI111711 PMC423500

[b4] Windler E. & Havel R. J. Inhibitory effects of C apolipoproteins from rats and humans on the uptake of triglyceride-rich lipoproteins and their remnants by the perfused rat liver. J. Lipid Res. 26, 556–565 (1985) . 4020294

[b5] Pollin T. I. *et al.* A null mutation in human APOC3 confers a favorable plasma lipid profile and apparent cardioprotection. Science 322, 1702–1705 (2008) . 19074352 10.1126/science.1161524 PMC2673993

[b6] Tachmazidou I. *et al.* A rare functional cardioprotective APOC3 variant has risen in frequency in distinct population isolates. Nat. Commun 4, 2872 (2013) . 24343240 10.1038/ncomms3872 PMC3905724

[b7] Crosby J. *et al.* Loss-of-function mutations in APOC3, triglycerides, and coronary disease. N. Engl. J. Med. 371, 22–31 (2014) . 24941081 10.1056/NEJMoa1307095 PMC4180269

[b8] Jorgensen A. B., Frikke-Schmidt R., Nordestgaard B. G. & Tybjaerg-Hansen A. Loss-of-function mutations in APOC3 and risk of ischemic vascular disease. N. Engl. J. Med. 371, 32–41 (2014) . 24941082 10.1056/NEJMoa1308027

[b9] Gangabadage C. S. *et al.* Structure and dynamics of human apolipoprotein CIII. J. Biol. Chem. 283, 17416–17427 (2008) . 18408013 10.1074/jbc.M800756200

[b10] Nichols W. C., Dwulet F. E., Liepnieks J. & Benson M. D. Variant apolipoprotein AI as a major constituent of a human hereditary amyloid. Biochem. Biophys. Res. Commun. 156, 762–768 (1988) . 3142462 10.1016/s0006-291x(88)80909-4

[b11] Benson M. D. *et al.* A new human hereditary amyloidosis: the result of a stop-codon mutation in the apolipoprotein AII gene. Genomics 72, 272–277 (2001) . 11401442 10.1006/geno.2000.6499

[b12] Sethi S. *et al.* Medullary amyloidosis associated with apolipoprotein A-IV deposition. Kidney Int. 81, 201–206 (2012) . 21900878 10.1038/ki.2011.316

[b13] de Messieres M., Huang R. K., He Y. & Lee J. C. Amyloid triangles, squares, and loops of apolipoprotein C-III. Biochemistry 53, 3261–3263 (2014) . 24804986 10.1021/bi500502d PMC4038341

[b14] Valleix S. *et al.* Hereditary systemic amyloidosis due to Asp76Asn variant beta 2-microglobulin. N. Engl. J. Med. 366, 2276–2283 (2012) . 22693999 10.1056/NEJMoa1201356

[b15] Vrana J. A. *et al.* Classification of amyloidosis by laser microdissection and mass spectrometry-based proteomic analysis in clinical biopsy specimens. Blood 114, 4957–4959 (2009) . 19797517 10.1182/blood-2009-07-230722

[b16] Naiki H., Higuchi K., Hosokawa M. & Takeda T. Fluorometric determination of amyloid fibrils in vitro using the fluorescent dye, thioflavin T1. Anal. Biochem. 177, 244–249 (1989) . 2729542 10.1016/0003-2697(89)90046-8

[b17] Liu H. *et al.* Characterization of the lipid-binding properties and lipoprotein lipase inhibition of a novel apolipoprotein C-III variant Ala23Thr. J. Lipid Res. 41, 1760–1771 (2000) . 11060345

[b18] Shachter N. S. Apolipoproteins C-I and C-III as important modulators of lipoprotein metabolism. Curr. Opin. Lipidol. 12, 297–304 (2001) . 11353333 10.1097/00041433-200106000-00009

[b19] Larsson M., Vorrsjo E., Talmud P., Lookene A. & Olivecrona G. Apolipoproteins C-I and C-III inhibit lipoprotein lipase activity by displacement of the enzyme from lipid droplets. J. Biol. Chem. 288, 33997–34008 (2013) . 24121499 10.1074/jbc.M113.495366 PMC3837139

[b20] Gregorini G. *et al.* Renal apolipoprotein A-I amyloidosis: a rare and usually ignored cause of hereditary tubulointerstitial nephritis. J. Am. Soc. Nephrol. 16, 3680–3686 (2005) . 16221867 10.1681/ASN.2005040382

[b21] Benson M. D., Liepnieks J., Uemichi T., Wheeler G. & Correa R. Hereditary renal amyloidosis associated with a mutant fibrinogen alpha-chain. Nat. Genet. 3, 252–255 (1993) . 8097946 10.1038/ng0393-252

[b22] Gillmore J. D. & Hawkins P. N. Pathophysiology and treatment of systemic amyloidosis. Nat. Rev. Nephrol 9, 574–586 (2013) . 23979488 10.1038/nrneph.2013.171

[b23] Pepys M. B. *et al.* Human lysozyme gene mutations cause hereditary systemic amyloidosis. Nature 362, 553–557 (1993) . 8464497 10.1038/362553a0

[b24] Maury C. P. Homozygous familial amyloidosis, Finnish type: demonstration of glomerular gelsolin-derived amyloid and non-amyloid tubular gelsolin. Clin. Nephrol. 40, 53–56 (1993) . 8395367

[b25] Marchesi M. *et al.* The intracellular quality control system down-regulates the secretion of amyloidogenic apolipoprotein A-I variants: a possible impact on the natural history of the disease. Biochim. Biophys. Acta 1812, 87–93 (2011) . 20637862 10.1016/j.bbadis.2010.07.002

[b26] Strauss J. G. *et al.* Adenovirus-mediated rescue of lipoprotein lipase-deficient mice. Lipolysis of triglyceride-rich lipoproteins is essential for high density lipoprotein maturation in mice. J. Biol. Chem. 276, 36083–36090 (2001) . 11432868 10.1074/jbc.M104430200

[b27] Jiang X. C. *et al.* Targeted mutation of plasma phospholipid transfer protein gene markedly reduces high-density lipoprotein levels. J. Clin. Invest. 103, 907–914 (1999) . 10079112 10.1172/JCI5578 PMC408146

[b28] von Eckardstein A. *et al.* Apolipoprotein C-III(Lys58----Glu). Identification of an apolipoprotein C-III variant in a family with hyperalphalipoproteinemia. J. Clin. Invest. 87, 1724–1731 (1991) . 2022742 10.1172/JCI115190 PMC295277

[b29] Epstein M. & Vaziri N. D. Statins in the management of dyslipidemia associated with chronic kidney disease. Nat. Rev. Nephrol 8, 214–223 (2012) . 22349484 10.1038/nrneph.2012.33

[b30] Holzer M. *et al.* Uremia alters HDL composition and function. J. Am. Soc. Nephrol. 22, 1631–1641 (2011) . 21804091 10.1681/ASN.2010111144 PMC3171935

[b31] Mange A. *et al.* HDL proteome in hemodialysis patients: a quantitative nanoflow liquid chromatography-tandem mass spectrometry approach. PLoS ONE 7, e34107 (2012) . 22470525 10.1371/journal.pone.0034107 PMC3309955

[b32] Capell W. H. *et al.* Short-term triglyceride lowering with fenofibrate improves vasodilator function in subjects with hypertriglyceridemia. Arterioscler. Thromb. Vasc. Biol. 23, 307–313 (2003) . 12588776 10.1161/01.atv.0000046230.02211.b4

[b33] Miller G. J. & Miller N. E. Plasma-high-density-lipoprotein concentration and development of ischaemic heart-disease. Lancet 1, 16–19 (1975) . 46338 10.1016/s0140-6736(75)92376-4

[b34] Kawakami A. & Yoshida M. Apolipoprotein CIII links dyslipidemia with atherosclerosis. J. Atheroscler. Thromb. 16, 6–11 (2009) . 19262004 10.5551/jat.e607

[b35] Haubenwallner S. *et al.* Hypolipidemic activity of select fibrates correlates to changes in hepatic apolipoprotein C-III expression: a potential physiologic basis for their mode of action. J. Lipid Res. 36, 2541–2551 (1995) . 8847480

[b36] Staels B. *et al.* Fibrates downregulate apolipoprotein C-III expression independent of induction of peroxisomal acyl coenzyme A oxidase. A potential mechanism for the hypolipidemic action of fibrates. J. Clin. Invest. 95, 705–712 (1995) . 7860752 10.1172/JCI117717 PMC295538

[b37] Coelho T. *et al.* Safety and efficacy of RNAi therapy for transthyretin amyloidosis. N. Engl. J. Med. 369, 819–829 (2013) . 23984729 10.1056/NEJMoa1208760

[b38] Boisfer E. *et al.* Overexpression of human apolipoprotein A-II in mice induces hypertriglyceridemia due to defective very low density lipoprotein hydrolysis. J. Biol. Chem. 274, 11564–11572 (1999) . 10206963 10.1074/jbc.274.17.11564

[b39] Chapman M. J., Goldstein S., Lagrange D. & Laplaud P. M. A density gradient ultracentrifugal procedure for the isolation of the major lipoprotein classes from human serum. J. Lipid Res. 22, 339–358 (1981) . 6787159

[b40] Guerin M., Bruckert E., Dolphin P. J., Turpin G. & Chapman M. J. Fenofibrate reduces plasma cholesteryl ester transfer from HDL to VLDL and normalizes the atherogenic, dense LDL profile in combined hyperlipidemia. Arterioscler. Thromb. Vasc. Biol. 16, 763–772 (1996) . 8640404 10.1161/01.atv.16.6.763

[b41] Guerin M. *et al.* Dose-dependent action of atorvastatin in type IIB hyperlipidemia: preferential and progressive reduction of atherogenic apoB-containing lipoprotein subclasses (VLDL-2, IDL, small dense LDL) and stimulation of cellular cholesterol efflux. Atherosclerosis 163, 287–296 (2002) . 12052475 10.1016/s0021-9150(02)00037-0

[b42] Kontush A., Chantepie S. & Chapman M. J. Small, dense HDL particles exert potent protection of atherogenic LDL against oxidative stress. Arterioscler. Thromb. Vasc. Biol. 23, 1881–1888 (2003) . 12920049 10.1161/01.ATV.0000091338.93223.E8

[b43] Camont L. *et al.* Small, dense high-density lipoprotein-3 particles are enriched in negatively charged phospholipids: relevance to cellular cholesterol efflux, antioxidative, antithrombotic, anti-inflammatory, and antiapoptotic functionalities. Arterioscler. Thromb. Vasc. Biol. 33, 2715–2723 (2013) . 24092747 10.1161/ATVBAHA.113.301468

[b44] Quehenberger O. & Dennis E. A. The human plasma lipidome. N. Engl. J. Med. 365, 1812–1823 (2011) . 22070478 10.1056/NEJMra1104901 PMC3412394

[b45] Larrede S. *et al.* Stimulation of cholesterol efflux by LXR agonists in cholesterol-loaded human macrophages is ABCA1-dependent but ABCG1-independent. Arterioscler. Thromb. Vasc. Biol. 29, 1930–1936 (2009) . 19729607 10.1161/ATVBAHA.109.194548

[b46] Fournier N. *et al.* Analysis of the relationship between triglyceridemia and HDL-phospholipid concentrations: consequences on the efflux capacity of serum in the Fu5AH system. Atherosclerosis 157, 315–323 (2001) . 11472731 10.1016/s0021-9150(00)00730-9

[b47] Kontush A., de Faria E. C., Chantepie S. & Chapman M. J. A normotriglyceridemic, low HDL-cholesterol phenotype is characterised by elevated oxidative stress and HDL particles with attenuated antioxidative activity. Atherosclerosis 182, 277–285 (2005) . 16159600 10.1016/j.atherosclerosis.2005.03.001

[b48] Nobecourt E. *et al.* Defective antioxidative activity of small dense HDL3 particles in type 2 diabetes: relationship to elevated oxidative stress and hyperglycaemia. Diabetologia 48, 529–538 (2005) . 15729582 10.1007/s00125-004-1655-5

[b49] Kontush A. & Chapman M. J. Antiatherogenic function of HDL particle subpopulations: focus on antioxidative activities. Curr. Opin. Lipidol. 21, 312–318 (2010) . 20581677 10.1097/MOL.0b013e32833bcdc1

[b50] Zerrad-Saadi A. *et al.* HDL3-mediated inactivation of LDL-associated phospholipid hydroperoxides is determined by the redox status of apolipoprotein A-I and HDL particle surface lipid rigidity: relevance to inflammation and atherogenesis. Arterioscler. Thromb. Vasc. Biol. 29, 2169–2175 (2009) . 19762782 10.1161/ATVBAHA.109.194555

[b51] Nicolardi S., van der Burgt Y. E., Wuhrer M. & Deelder A. M. Mapping O-glycosylation of apolipoprotein C-III in MALDI-FT-ICR protein profiles. Proteomics 13, 992–1001 (2013) . 23335445 10.1002/pmic.201200293

[b52] Hawkins P. N., Lavender J. P. & Pepys M. B. Evaluation of systemic amyloidosis by scintigraphy with 123I-labeled serum amyloid P component. N. Engl. J. Med. 323, 508–513 (1990) . 2377176 10.1056/NEJM199008233230803

[b53] Garnier J., Gibrat J. F. & Robson B. GOR method for predicting protein secondary structure from amino acid sequence. Methods Enzymol. 266, 540–553 (1996) . 8743705 10.1016/s0076-6879(96)66034-0

[b54] Tartaglia G. G. *et al.* Prediction of aggregation-prone regions in structured proteins. J. Mol. Biol. 380, 425–436 (2008) . 18514226 10.1016/j.jmb.2008.05.013

[b55] De Pauw M., Vanloo B., Weisgraber K. & Rosseneu M. Comparison of lipid-binding and lecithin:cholesterol acyltransferase activation of the amino- and carboxyl-terminal domains of human apolipoprotein E3. Biochemistry 34, 10953–10966 (1995) . 7662677 10.1021/bi00034a030

[b56] Bengtsson-Olivecrona G. & Olivecrona T. In Lipoprotein Analysis-A Practical Approach Oxford Univ. (1992) .

