# Supplementary material for: D25V apolipoprotein C-III variant causes dominant hereditary systemic amyloidosis and confers cardiovascular protective lipoprotein profile
Source: Nat Commun. 2016 Jan 21;7:10353. doi: 10.1038/ncomms10353 (PMC4735822; doi:10.1038/ncomms10353)
Supplement: Supplementary Information — Supplementary Figures 1-2 and Supplementary Tables 1-2 [file ncomms10353-s1.pdf]

# SUPPLEMENTARY INFORMATION

## SUPPLEMENTARY FIGURES

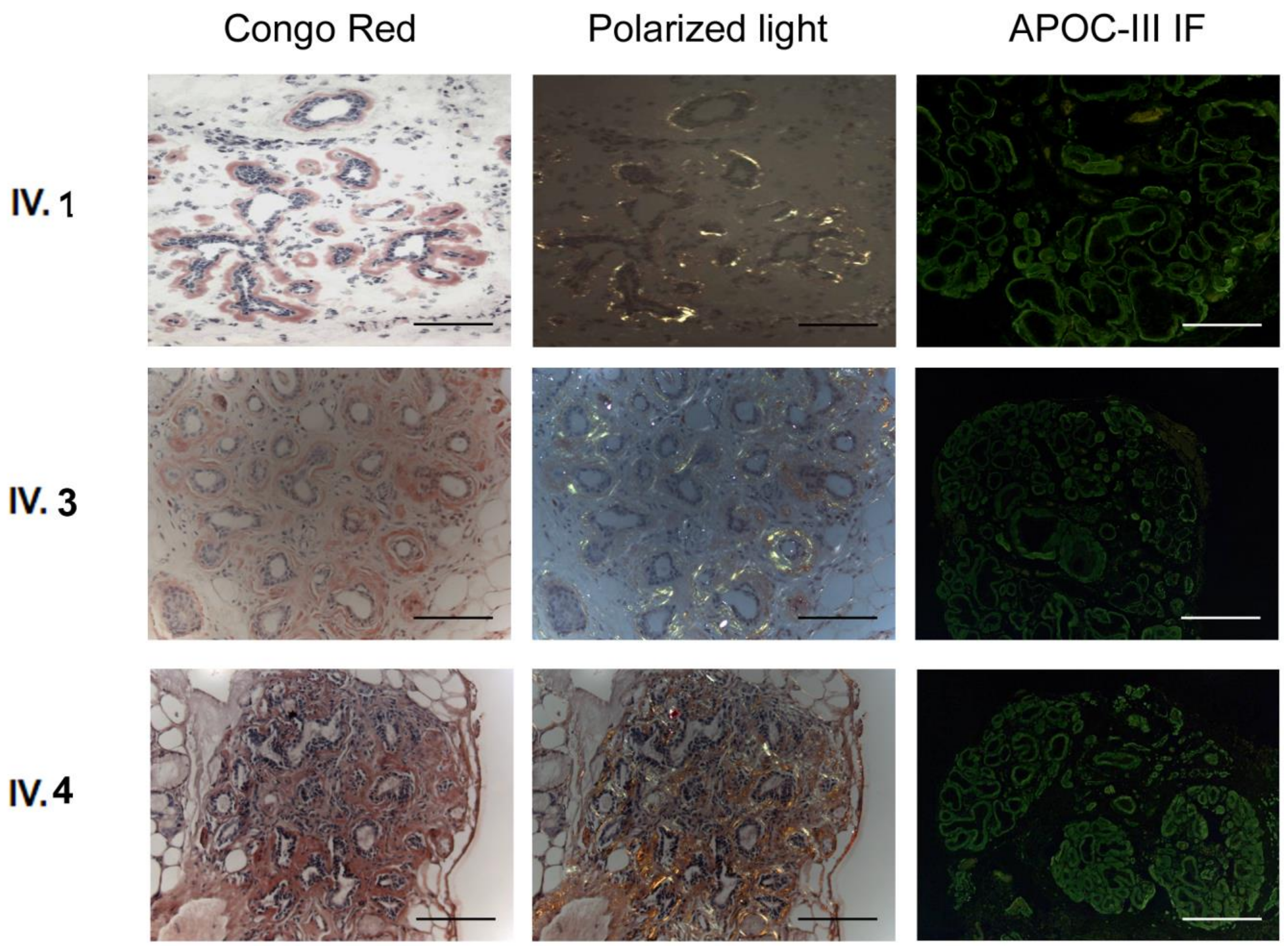


**Supplementary Fig. 1. Histology of salivary gland tissue.** Amyloid deposits in the salivary gland tissue from three additional D25V-carriers IV.1, IV.3 stained with Congo red, exhibited green birefringence when viewed under polarized light and were immunoreactive for apoC-III antibody in all D25V-carriers. Scale bar: 100 µm.

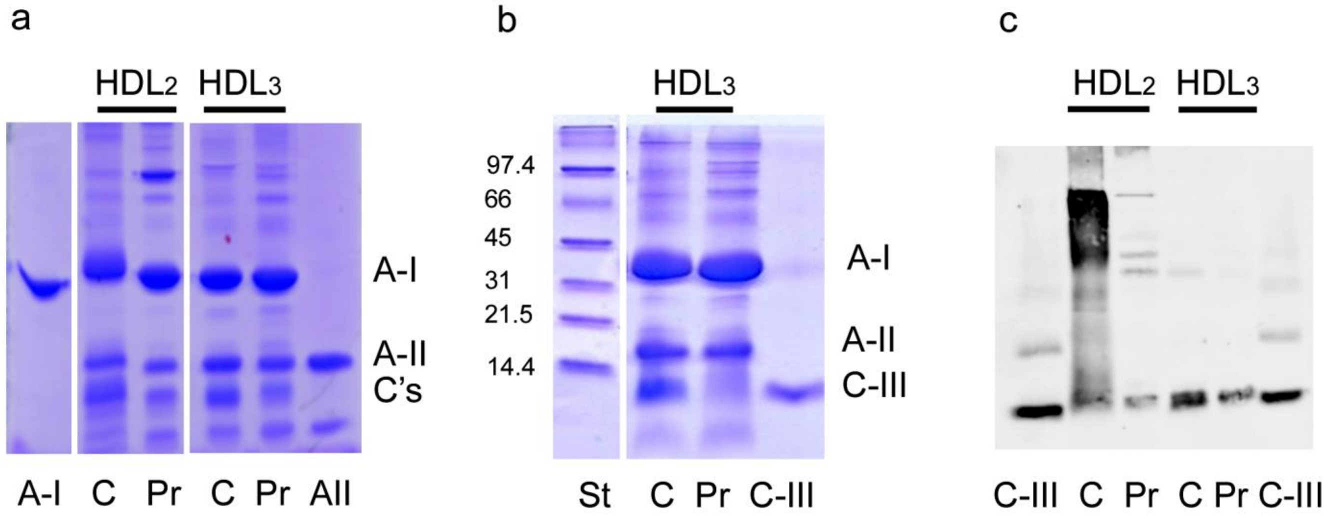


**Supplementary Fig. 2. Analysis of HDL apolipoproteins.** (**a**) SDS 15% PAGE of $HDL_2$ and $HDL_3$ from the proband III.3 and the control subject (12 µg protein per lane) carried out under reducing conditions. In the first and last lane, respectively, purified human apoA-I (28 kDa) and human dimeric apoA-II (17 kDa), the major HDL apolipoproteins, were run as standards. (**b**) SDS 15% PAGE of HDL3 from the proband and the control subject (12 µg protein per lane) was carried out as in (a). Low Range SDS-PAGE Standards (kDa) (LMW BioRad) were run in the first lane, and purified human apoC-III in the last lane as standard (4 µg per lane). SDS-PAGE for HDL2 and HDL3 were replicated 7 times, using as standards either purified apoA-I, apoA-II and apoC-III or LMW. (**c**) Western blot developed with a primary rabbit anti-human apoC-III antibody (1 µg $ml^{-1}$, Abnova). Isolated apoC-III (8.8 kDa, 4 µg per lane) was loaded in the first and last lanes as standard controls. Western blots for apoC-III were replicated 3 times. A-I, apolipoprotein A-IC, control subject; Pr, proband III.3; A-II, apolipoprotein AII; C-III, apolipoprotein C-III.

**Supplementary Table 1. Amyloid proteome of salivary gland.**

| Entry Name (a) | Accession number (b) | MW (kDa) | Sample 1 | Sample 2 |
|---|---|---|---|---|
| Serum albumin | ALBU_HUMAN | 69 | 22 | 20 |
| Collagen alpha-3(VI) chain | CO6A3_HUMAN | 34 | 17 | 16 |
| **Apolipoprotein E** | **APOE_HUMAN** | 36 | 17 | 13 |
| Keratin, type I cytoskeletal 10 | K1C10_HUMAN | 58 | 15 | 14 |
| Lactotransferrin | TRFL_HUMAN | 78 | 15 | 14 |
| **Apolipoprotein C-III** | **APOC3_HUMAN** | 11 | 13 | 8 |
| Keratin, type II cytoskeletal 2 epidermal | K22E_HUMAN | 65 | 12 | 10 |
| Keratin, type II cytoskeletal 1 | K2C1_HUMAN | 66 | 11 | 10 |
| Vitronectin | VTNC_HUMAN | 54 | 10 | 9 |
| **Serum amyloid P-component** | **SAMP_HUMAN** | 25 | 10 | 9 |
| Vimentin | VIME_HUMAN | 54 | 9 | 9 |
| Basement membrane-specific heparan sulfate proteoglycan core protein | PGBM_HUMAN | 47 | 8 | 8 |
| Keratin, type I cytoskeletal 19 | K1C19_HUMAN | 44 | 8 | 8 |
| Hemoglobin subunit beta | HBB_HUMAN | 16 | 8 | 7 |
| Keratin, type II cytoskeletal 7 | K2C7_HUMAN | 51 | 6 | 6 |
| Apolipoprotein A-IV | APOA4_HUMAN | 45 | 6 | 6 |
| Keratin, type II cytoskeletal 5 | K2C5_HUMAN | 62 | 6 | 6 |
| Keratin, type I cytoskeletal 14 | K1C14_HUMAN | 51 | 5 | 5 |
| Collagen alpha-2(VI) chain | CO6A2_HUMAN | 11 | 4 | 4 |
| Actin, cytoplasmic 1 | ACTB_HUMAN | 42 | 4 | 3 |
| Complement component C9 | CO9_HUMAN | 65 | 3 | 2 |

Amyloid proteome of salivary gland from individual IV.3 detected apoC-III protein along with apoE and SAP.

**Supplementary Table 2. Primer sequences for PCR amplification of *APOC3*.**

| PRIMER SEQUENCES FOR PCR AMPLIFICATION OF THE *APOC3* GENE | | | |
|---|---|---|---|
| | Primer name | Primer sequence (5'->3') | Product size (bp) |
| Promoter region | *APOC3_5PF* | gaaggtgaacgagagaatcagtcctg | 511 |
| | *APOC3 _5PR* | gcctcgggcccatctcagcctttcacactg | |
| Exon 1 | *APOC3_1F* | caagccacccacttgttctc | 600 |
| | *APOC3 _1R* | tccgaggcttccttagctc | |
| Exon 2 | *APOC3_2F* | cttctggcagacccagctaa | 250 |
| | *APOC3 _2R* | gaccacccattgggactg | |
| Exon 3 | *APOC3 _3F* | atgggtggtcaagcagga | 296 |
| | *APOC3 _3R* | gagcacctccattccattgt | |
| Exon 4 | *APOC3 _4F* | ctgactggtgtcgtccagtg | 288 |
| | *APOC3 _4R* | ccctggagattgcaggac | |
| 3'UTR | *APOC3_3PF* | ggagcaccgttaaggacaag | 562 |
| | *APOC3_3PR* | ggggcacacactcaagtcat | |
